# Identification of microbial communities associated with *Phymatotrichopsis omnivora* sclerotia in two Texas fields

**DOI:** 10.3389/frmbi.2025.1666691

**Published:** 2025-11-28

**Authors:** Maxwell Sturdivant, Sanjay Antony-Babu, Elizabeth Pierson, Thomas M. Chappell, Thomas Isakeit

**Affiliations:** 1 Department of Plant Pathology and Microbiology, Texas A&M University, College, Station, TX, United States; 2 Department of Horticultural Sciences, Texas A&M University, College, Station, TX, United States

**Keywords:** *Phymatotrichopsis omnivora*, sclerotia-associated microorganisms, cotton, *Gossypium hirsutum*, cotton root rot

## Abstract

The soilborne fungus *Phymatotrichopsis omnivora* causes a mid- to late-season disease known as cotton root rot (CRR). In the United States, *P. omnivora* is primarily found in Arizona, New Mexico, Oklahoma, and Texas in soils that are alkaline, calcareous, and rarely freeze deeply. This fungus has a wide host range, and can cause substantial losses in cotton crops. In Texas, not all cotton-producing soils have widespread CRR despite having the characteristics to support *P. omnivora*. Considering the lack of CRR in some Texas soils, we hypothesize that this absence could be due to the microbial composition associated with sclerotia of *P. omnivora*. The objective of this study was to identify the taxa that make up microbial communities associated with *P. omnivora* sclerotia in different soils during both the cotton-growing and off seasons. The microbiota associated with *P. omnivora* sclerotia were identified by burying lab-generated sclerotia in cotton-producing soils. These sclerotia were recovered, along with soil samples for metabarcoding targeting the 16S rRNA gene and the internal transcribed spacer region. When compared to bulk soil, microbial communities associated with sclerotia differed in community composition and taxa relative abundance between a soil with widespread CRR and one in which the disease is absent. Within these soil communities, potential bacterial and fungal biomarkers that reduce CRR were identified. Furthermore, microbial communities of *P. omnivora* sclerotia changed seasonally. This study presents the first detailed characterization of microorganisms associated with *P. omnivora* sclerotia in different cotton-producing soils. Our findings support the view that *P. omnivora* sclerotia serve as ecological hubs, shaping microbial communities with possible implications for disease suppression. Several enriched taxa are culturable, offering candidates for future biocontrol studies that could inform disease management strategies that focus on increased microbial competition.

## 1 Introduction

Cotton root rot (CRR), caused by the soilborne fungal plant pathogen *Phymatotrichopsis omnivora* (Duggar) Hennebert 1973, is a mid- to late-season disease affecting economically important crops in the American southwest such as cotton, pecan trees, winegrape, and alfalfa. *P. omnivora* forms sclerotia in soils from southwestern Arkansas, through the majority of Texas, to southern Arizona (Streets and Bloss, 1973). Annual losses from CRR in Texas cotton can reach $29 million in years that are especially conducive to disease progression (Isakeit, 2021), and in the 2024 growing season, an estimated 20,026 bales were lost to CRR in the southwestern cotton crop (Faske and Sisson, 2025). Even though CRR can have a significant impact in Texas, the disease is not present in all parts of the state, including cotton-producing soils with the characteristics to support the pathogen, specifically, alkaline, calcareous, and rarely freezing deeply (Percy, 1983). We hypothesize that the absence of CRR could be due to a suppressive microbial composition. The relationship between *P. omnivora* and other soil microorganisms has been of interest for many years. A study by Chavez et al. (1976) showed that increased microbial diversity, through the addition of green manure to a field, can reduce the incidence of CRR. Furthermore, sclerotia of *P. omnivora* support bacterial communities that include fluorescent pseudomonads and actinomycetes (Zuberer et al., 1988). There are several examples of soil microbiomes mediating plant disease including microbial richness and diversity affecting fusarium wilt in vanilla (*Vanilla* planifolia; F*. oxysporum* f. sp. *vanilla*) and banana (*Musa* species; *F. oxysporum* f. sp. *cubense* Tropical Race 4) (Xiong et al., 2017; Jamil et al., 2023), community composition affecting potato scab (*Streptomyces* species) incidence (Shi et al., 2019), populations of Firmicutes and Actinobacteria in the tomato rhizosphere protecting the plant against bacterial wilt (*Ralstonia solanacearum*) (Lee et al., 2021), and rhizosphere selection over successive wheat plantings affecting root disease caused by *Rhizoctonia solani* (Yin et al., 2021).

Fairly recently, the concept of the pathobiome has emerged as a compelling area of study. Pathogen-associated microorganisms can affect the ability of that pathogen to infect a host, or even create a scenario in which a commensal microbe turns pathogenic due to a change in the microbiome (Vayssier-Taussat et al., 2014). The term for microbes associated with pathogens is “pathobionts”, and they can have a great impact on disease (Jochum and Stecher, 2020). Some pathobionts live within fungal partners as endohyphal bacteria, influencing the virulence of fungal plant pathogens, as well as contributing to the survival of fungi through the modulation of hormones and metabolites (Moses and Carter, 2025). Obasa et al. (2019) demonstrated that two endohyphal strains of *Enterobacter* increased macroconidia and fumonisin production in *F. fujikuroi*, leading to higher levels of virulence. Thomas and Antony-Babu (2024) identified the core bacterial hyphosphere of a Fusarium wilt pathogen, *F. oxysporum* f. sp. *niveum*, which shed light on pathobionts contributing to virulence, and strengthens the idea that pathogens are not lone invaders when they gain access to a host. This work was expanded upon by Thomas et al. (2025) when it was shown that the microbial community structure of the *F. oxysporum* f. sp. *cubense* tropical race 4 hyphosphere was highly correlated with the microbial community structure of diseased tissue colonized by the pathogen. Additionally, Jakuschkin et al. (2016) investigating microbial interactions on oak leaves (*Quercus robur* L.), found that oak powdery mildew (*Erysiphe alphitoides*) is both positively and negatively associated with discrete operational taxonomic units (OTUs) in the phyllosphere. Looking to cotton as a system for the study of bacterial-fungal interactions, Antony-Babu et al. (2025) showed that members of the genus *Pseudomonas* were preferentially selected for in the *F. oxysporum* f. sp. *vasinfectum* Race 4 (FOV4) hyphosphere, a fungus that causes Fusarium wilt in cotton. This finding has implications for virulence and survival of FOV4 because several *Pseudomonas* isolates that were associated with hyphal tips significantly promoted hyphal growth.

In addition to contributing to fungal virulence, there are also bacterial members of the pathobiome that feed on fungi, exhibiting mycophagy. Bacterial mycophagy can be defined as a set of behaviors that allow bacteria to convert fungal biomass into a nutrient source (de Boer et al., 2005; Fritsche et al., 2006). These mycophagous bacteria have implications for suppressive soils if they are able to feed on plant pathogenic fungi. Mycophagous bacteria exhibit three strategies to derive nutrients from fungi: necrotrophy, extracellular biotrophy, or endocellular biotrophy (Leveau and Preston, 2008). These strategies demonstrate that bacteria feeding on a fungus does not mean that they are killing that organism, which also has biocontrol implications. A pathobiome study has not been done with the primary inoculum of *P. omnivora*, the sclerotia. Furthermore, the microbial ecology of fungal sclerotia generally is an understudied field. As previously mentioned, there is evidence to suggest that there is a microbial aspect in the success of *P. omnivora* to cause CRR.

In this ecological study, soil microorganisms were identified using metabarcoding to better understand the microbial community associated with *P. omnivora* sclerotia. The core hypothesis of this study is that live sclerotia will deterministically recruit a functional microbial community. With this hypothesis in mind, the objectives of this study were to identify microbial communities associated with *P. omnivora* sclerotia in fields that differ in their of support CRR during the cotton-growing and off seasons, as well as determine if these communities change between seasons. To investigate these objectives, metabarcoding was used to sequence soil in contact with lab-grown sclerotia. One cycle of seasonal change was evaluated at two locations with contrasting histories of CRR. Additionally, changes in microbial communities were assessed over three seasons in a soil that is CRR-conducive. Finally, potential determinism in the sclerotia-associated communities was assessed using linear discriminant analysis effect size (LEfSe) and Beta-Nearest Taxon Index (βNTI) to identify biomarkers in microbial communities associated with particular niches and assess deterministic selection within those niches.

## 2 Materials and methods

### 2.1 *P. omnivora* isolates and test sites

A single *P. omnivora* isolate was used in each of the burial seasons. For the 2023 and 2024 cotton-growing seasons, an isolate obtained from Willacy County, TX in 2022 was used. For the 2023–2024 off-season, an isolate obtained from Williamson County, TX in 2023 was used. *P. omnivora* isolates used in this study were collected from infected upper root tissue of cotton plants (*Gossypium hirsutum*) by thoroughly washing with water, followed by copious spraying with 70% ethanol and repeated whittling and peeling of outer tissue, until a lesion was identified. From this lesion, small pieces of tissue were excised and plated on half-strength potato dextrose agar amended with 10 mg of streptomycin per 100 mL (½ PDAs). The advancing margins of developing growth of *P. omnivora* were then transferred to a new ½ PDAs plate to obtain a pure culture. All isolates were stored at ambient room temperature (22°C) and sub-cultured monthly to maintain viability. These isolates were used to generate sclerotia as described in Section 3.2.

Lab-generated sclerotia were buried in one location during the 2023 cotton-growing season, as well as two locations during the 2023–2024 off-season and the 2024 cotton-growing season. The first location was the Stiles Farm in Williamson County, TX, and is considered the CRR-conducive location because there is widespread CRR in that field. The soil type at the location of the Stiles Farm where the sclerotia were buried is a Burleson clay (USDA NRCS). Its characteristics were 30% sand, 30% silt, 40% clay, pH 7.7, 1.7% organic matter, and a cation exchange capacity (CEC) of 33. Sclerotia were buried at this location during all three seasons. The second location was the Texas A&M Research Farm (Bottom Farm) in Burleson County, TX, and this location is considered to be non-conducive to CRR because the disease does not occur in that soil type in that area. The soil type at the location of the Bottom Farm where the sclerotia were buried is a Weswood silty clay loam (USDA NRCS). Its characteristics were 19% sand, 55% silt, 26% clay, pH 8.0, 1.2% organic matter, and a CEC of 30. Sclerotia were buried in this location during the 2023–2024 off-season and the 2024 cotton-growing season. Sclerotia were buried in mesh bags from June 2023 to September 2023 for the 2023 cotton-growing season, from December 2023 to March 2024 for the off-season burial period, and from June 2024 to September 2024 for the 2024 cotton-growing season burial period.

### 2.2 Sclerotia preparation in laboratory microcosms

Sclerotia were produced *in vitro* in a sorghum seed-soil substrate following a modification of the protocol of Dunlap (1941). Each microcosm was prepared in 250 mL Erlenmeyer flasks containing 10g of autoclaved sorghum seed and 100g of air-dried soil that was wetted with 30 mL reverse-osmosis (RO) water and inoculated with an agar plug of *P. omnivora*. The sorghum seed layer, above the soil, served as a growth medium for *P. omnivora*, from which it produced sclerotia in soil from rhizomorphs. The seed, initially in glass Petri dishes, along with 5 mL RO water, was autoclaved on a gravity cycle (121°C, 24 PSI) for 20 min. After autoclaving, the sorghum seed was placed on top of the soil within the Erlenmeyer flask that had been moistened with RO water. This flask was then autoclaved on a gravity cycle for one hour, allowed to cool, then inoculated with an agar plug of *P. omnivora*. These inoculated flasks were placed in plastic bags and incubated at 32°C for five weeks. At that time, the colonized sorghum was removed from the flasks and discarded, while the soil was wet sieved over a number 18 sieve (1 millimeter opening). The sclerotia were collected from the sieve, surface sterilized in a 10% bleach solution for 30 seconds, then rinsed in autoclaved RO water for 1 min. The sclerotia were quickly blotted dry on sterile filter paper then transferred to a glass Petri dish containing autoclaved soil for storage. This Petri dish was sealed with Parafilm and stored at ambient room temperature.

### 2.3 Field inoculation of *P. omnivora* sclerotia

To assess the microbial communities associated with *P. omnivora* sclerotia, we deployed laboratory-generated sclerotia using retrievable mesh bags. The mesh bag setup consisted of a firm plastic 1-mm^2^ opening mesh, 9.5 x 6 mm in size, with an opening at the top, which contained 10–15 sclerotia. The sclerotia bags were deployed across two geographically distinct sites in Texas: (1) the Stiles Farm in Williamson County, a site with widespread CRR and thus considered CRR-conducive, where sclerotia were buried during the 2023 cotton-growing season, the 2023–2024 off-season, and the 2024 cotton-growing season; and (2) the Texas A&M Research Farm (Bottom Farm) in Burleson County, a CRR-nonconducive site based on the absence of disease in the local soil type, where sclerotia were buried during the 2023–2024 off-season and 2024 growing season. There were a total of nine bags buried about 15 cm deep in three rows. The heat-killed sclerotia were autoclaved prior to burial. The burial locations of the bags were marked with stakes. Viability of sub-samples of sclerotia used in the burials was tested on ½ PDAs and unamended nutrient agar prior to burial, and germination of the recovered sclerotia was tested on ½ PDAs.

### 2.4 Soil collection

Following the three-month field incubation periods, the bags were recovered, and soil in contact with the bags was collected. Bulk soil samples were collected from two locations approximately 15 cm deep and at least 6 m away from any sclerotia bag, to serve as controls. One soil sample from each bag burial and bulk soil location was collected in a 50 mL centrifuge tube using plastic spoons for a total of 11 soil samples (six live sclerotia, three heat-killed sclerotia, and two bulk soil). These samples were transported back to the laboratory in a cooler with dry ice and then stored at 2 to 8°C prior to DNA extraction.

### 2.5 DNA extraction and metabarcoding sequencing

For DNA extraction, two preparations were taken from each soil sample providing 22 soil DNA samples (12 live sclerotia, six heat-killed sclerotia, and four bulk soil). DNA extraction was done using the DNeasy® PowerSoil® Pro Kit (Qiagen, Valencia, CA, USA) following the protocol provided by the manufacturer. Extracted DNA quality was evaluated using a Quickdrop Micro-volume spectrophotometer (Molecular Devices, San Jose, CA, USA). DNA was quantified using a Qubit Double-Stranded DNA High-Sensitivity Assay Kit (Thermo Fisher Scientific, MA, USA) in a Qubit 2.0 fluorometer (Invitrogen, CA, USA). Library preparation and sequencing was done through Novogene (Sacramento, CA, USA). Amplicon libraries were prepared using the primers 341F – CCT AYG GGR BGC ASC AG and 806R – GGA CTA CNN GGG TAT CTA AT targeting the V3-V4 region of 16S rRNA. For ITS region-based library preparation, the primers ITS5-1737-F – GGA AGT AAA AGT CGT AAC AAG G and ITS2-2043- R – GCT GCG TTC TTC ATC GAT GC were used. Twenty-two separate samples were sequenced for both bacterial and fungal identification, totaling 44 samples per burial location for each of the seasons.

### 2.6 Metabarcoding sequence analysis pipeline

Raw reads were processed using Mothur v.1.48 (Schloss et al., 2009). Mothur was run utilizing computing resources from the Texas A&M High Performance Research Computing Department. The protocols for Mothur were followed based on the MiSeq standard operating procedure (https://mothur.org/wiki/miseq_sop/). The total number of reads for the 2023 cotton-growing season was 1,036,501 for 16S V3-V4 amplicon sequences and 510,923 for ITS region amplicon sequences after quality filtering. For ITS region analysis during the 2023 cotton-growing season, sequence reads exceeding 400 base pairs (bp) were removed. The total number of reads for the 2023–2024 off-season was 2,336,959 for 16S V3-V4 amplicon sequences and 876,704 for ITS region amplicon sequences after quality filtering. The total number of reads for the 2024 cotton-growing season was 2,318,350 for 16S V3-V4 amplicon sequences and 1,369,654 for ITS amplicon sequences after quality filtering. Additionally, any ambiguous bases and maximum repeats for eight or more nucleotide sequences were removed using “maxambig” and “maxhomop” in Mothur. Mothur functions were applied to make contigs, filter out bad reads, remove chimeras, and assign OTUs. A 97% cutoff was applied to bacterial and fungal taxa. For taxonomic assignment of 16S rRNA reads, the Ribosomal Database project classifier (Wang et al., 2007) with the Silva database v138 (Quast et al., 2013) was used. For taxonomic assignment of ITS region reads, a Mothur release of UNITE database v6 (Abarenkov et al., 2021) was used. Using “remove.lineage” in Mothur, OTUs assigned to chloroplast, mitochondria, Archaea, Eukaryota, and unknown were removed for 16S rRNA reads, and OTUs assigned to chloroplast, mitochondria, Archaea, and unknown were removed for ITS region reads.

### 2.7 Data analysis and statistical evaluation

Mothur was used to perform an analysis of molecular variance (AMOVA) to compare sclerotia treatments to each other and to bulk soil. Files created in Mothur were input into RStudio utilizing multiple R versions due to the extended time frame of data analysis. In RStudio, the packages “tidyverse” and “broom” were used for statistical analysis and data visualization. Soil microbial diversity was assessed using the Shannon and inverse Simpson diversity indices, observed diversity, and evenness, and statistical comparisons were made using a t-test. Comparison of diversity between locations and seasons, or beta-diversity, was visualized using non-metric multidimensional scaling (NMDS) constructed using the Bray-Curtis dissimilarity matrix, with significant differences determined using the AMOVA results from Mothur. Differences in relative abundance of taxa within a location or season, or alpha-diversity, were assessed using a pairwise Wilcoxon test. To assess which OTUs were enriched when comparing sclerotia treatments, locations, and seasons, LEfSe analyses were performed to identify biomarkers within microbial communities (Segata et al., 2011). To evaluate for deterministic selection in microbial communities, βNTI analyses were performed utilizing the “microeco” package in RStudio (Liu et al., 2021). βNTI analyses reveal whether the phylogenetic composition of communities are more similar or dissimilar than expected under stochastic assembly models. Phylogenetic trees used in the βNTI analyses were created with FASTA outputs from “get.oturep” in mothur to generate a list of OTUs and representative sequences. For all analyses, differences were considered significant at an alpha level of 0.05, unless otherwise noted.

## 3 Results

### 3.1 Comparison of communities between locations: 2023–2024 off-season

**Figure 1:**
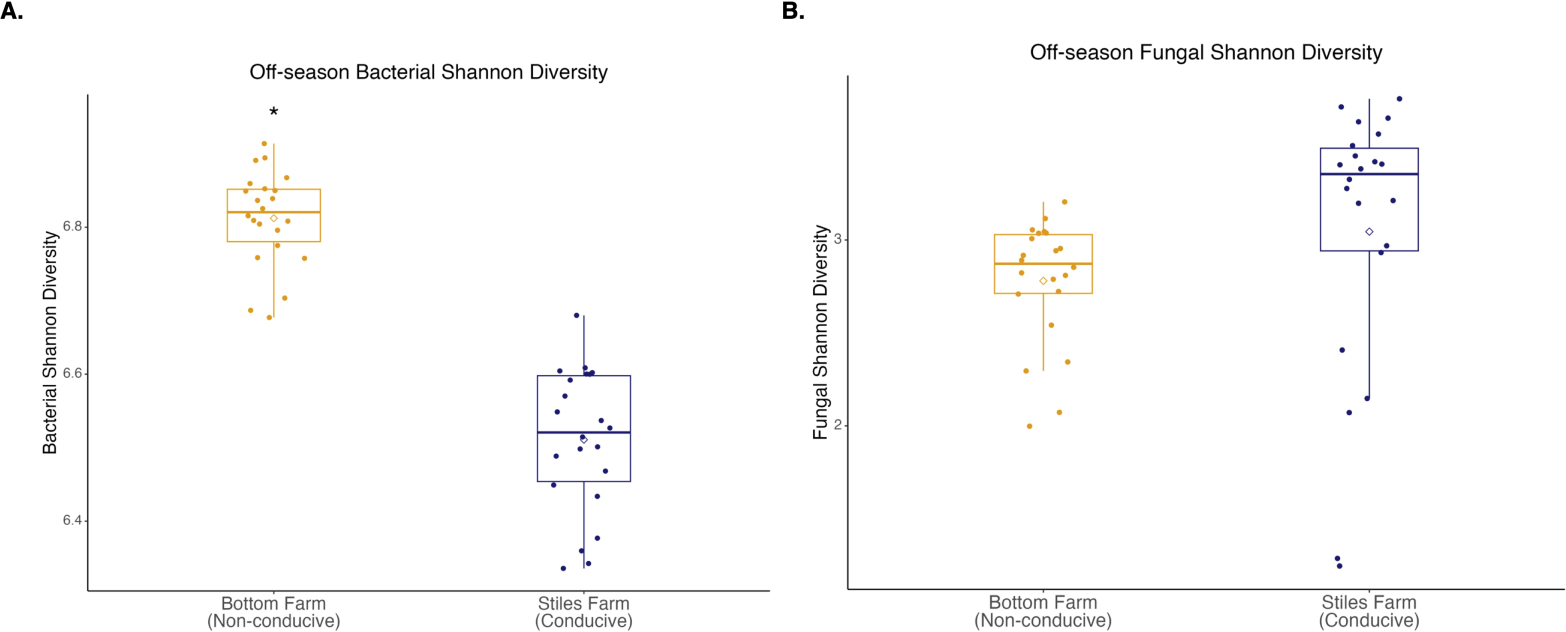
Microbial diversity during the 2023–2024 off-season. **(A)** Bacterial Shannon diversity was significantly (p<0.001) higher at the Bottom Farm. **(B)** Fungal Shannon diversity was numerically higher at the Stiles Farm, but not significantly. “*” represents a significant difference.

**Figure 2:**
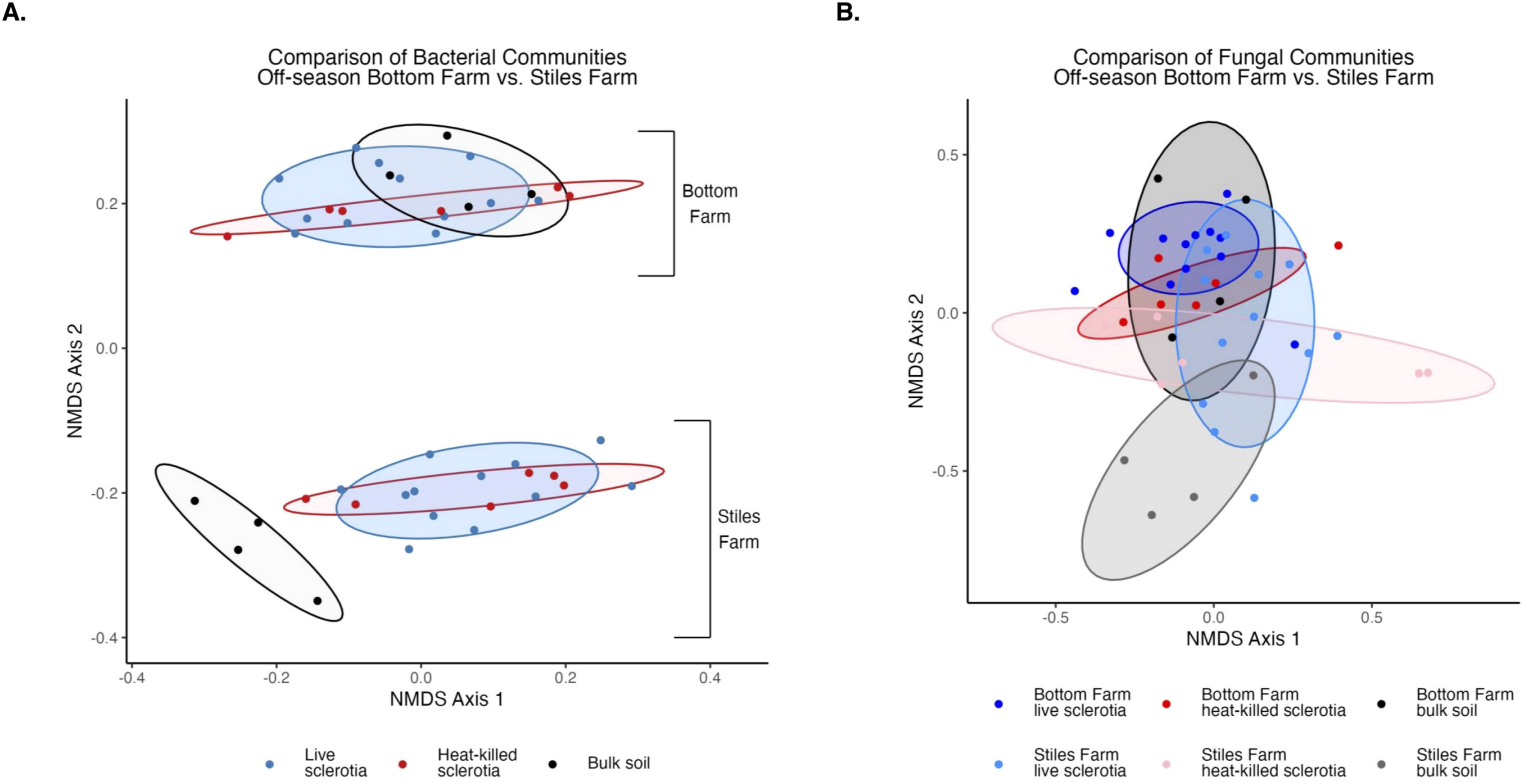
Comparison of microbial communities between locations during the 2023–2024 off-season separated by sclerotia treatment. Bacterial **(A)** and fungal **(B)** communities were significantly (p<0.001) different by location.

Comparing the two soils, based on the Shannon Index, bacterial diversity was significantly (p<0.001) higher at the CRR-nonconducive Bottom Farm (**Figure 1A**), while fungal diversity was numerically higher at the CRR-conducive Stiles Farm (**Figure 1B**). When comparing the diversity of the communities between the soils, both bacterial (Fs=8.8, p<0.001) and fungal (Fs=6.3, p<0.001) communities significantly differed from one another (**Figure 2**). For each location, sclerotia and bulk soil treatments were significantly different. At the Bottom Farm, bacterial communities associated with live sclerotia differed significantly from those in bulk soil (p<0.001), as did communities associated with heat-killed sclerotia (p=0.049). Similarly, at the Stiles Farm, both live sclerotia (p<0.001) and heat-killed sclerotia (p=0.005) supported bacterial communities that were significantly different from the bulk soil. However, no significant differences were observed between bacterial communities associated with live and heat-killed sclerotia at either site. The fungal communities followed a similar pattern except for the Bottom Farm, where no significant differences were detected among treatments. At the Stiles Farm, fungal communities associated with both live sclerotia (p=0.007) and heat-killed sclerotia (p=0.043) differed significantly from the bulk soil, while no significant differences were observed between live and heat-killed sclerotia at either site.

**Figure 3:**
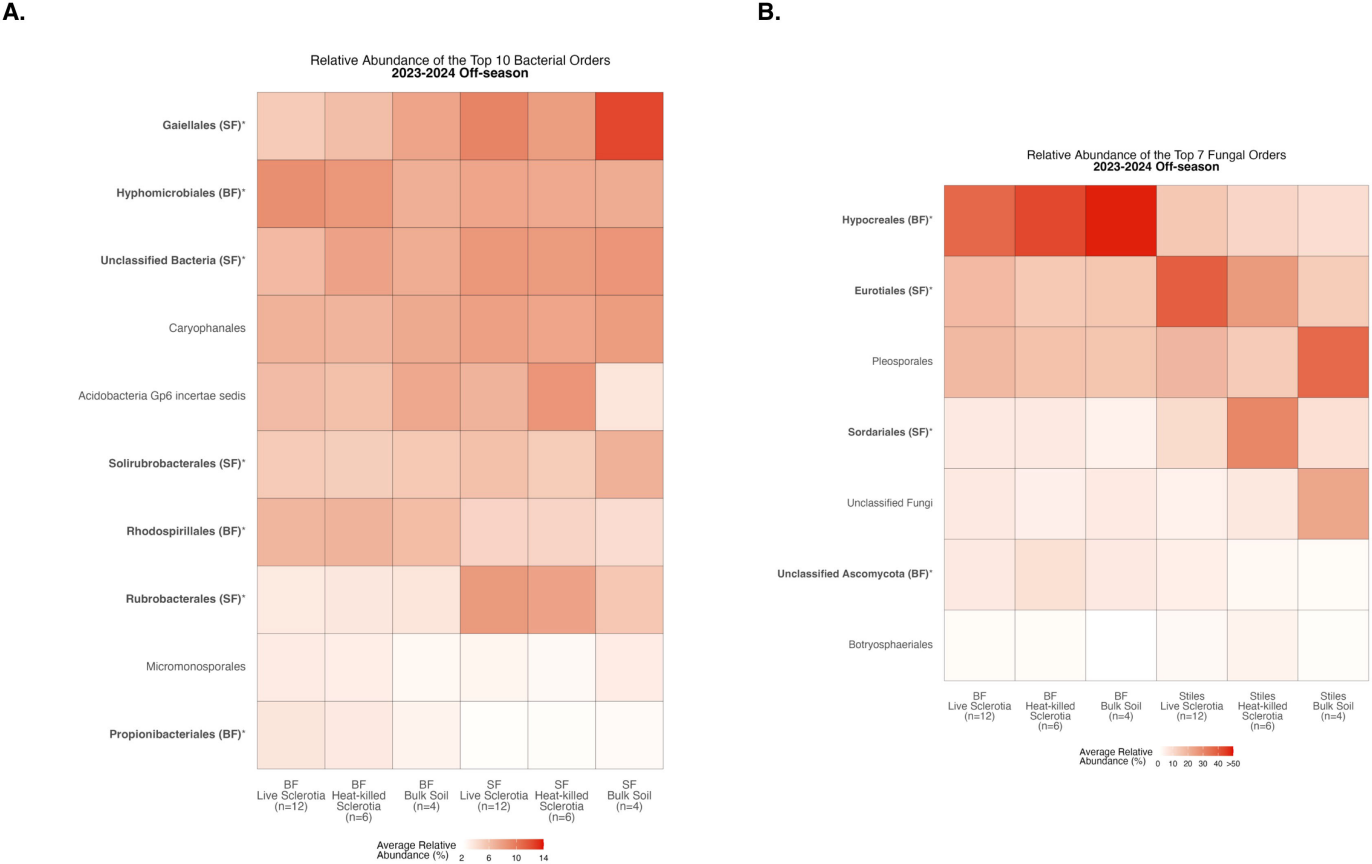
Heatmaps displaying diversity within location during the 2023–2024 off-season. Bolded orders are significantly (p<0.05) different between the Bottom Farm (BF) and Stiles Farm (SF). “(BF)” or “(SF)” after a bolded order indicates which location had a significantly higher relative abundance of that order. **(A)** Comparison of bacterial communities. **(B)** Comparison of fungal communities.

To assess compositional differences, we identified the 10 most abundant bacterial orders averaged across samples in each of the sclerotia treatments and bulk soil (**Supplementary Table S1**). At the Bottom Farm, bacteria affiliated with the orders *Hyphomicrobiales*, *Rhodospirillales*, and *Propionibacteriales* were found at a significantly higher relative abundance than at the Stiles Farm (**Figure 3A**). At the Stiles Farm, bacteria in the orders *Gaiellales*, Unclassified Bacteria, *Solirubrobacterales*, and *Rubrobacterales* were found at a significantly higher relative abundance than at the Bottom Farm (**Figure 3A**). With fungal communities, there were significant differences among the seven most abundant fungal orders averaged across samples in each of the sclerotia treatments and bulk soil (**Supplementary Table S2**). At the Bottom Farm, fungal orders *Hypocreales* and Unclassified Ascomycota were found at a significantly higher relative abundance than at the Stiles Farm (**Figure 3B**). However, at the Stiles Farm, fungi in the orders *Eurotiales* and *Sordariales* were found at a significantly higher relative abundance than at the Bottom Farm (**Figure 3B**). The full breadth of bacterial and fungal alpha diversity during the 2023–2024 off-season, at the order level, are presented in **Supplementary Figures S5**, **S6**, respectively.

In the Bottom Farm communities, the genus *Pseudomonas* made up 0.06% of the community, while members of the class Actinobacteria made up 17.86%. In the Stiles Farm communities, the genus *Pseudomonas* made up 0.18% of the community, while members of the class Actinobacteria made up 13%.

Based on the results of three LEfSe analyses, genera that were enriched in live sclerotia treatments when compared to bulk soil, but not enriched in heat-killed sclerotia, were selected. At the Bottom Farm, seven bacterial genera met these criteria (with the percentage that genus represents in the live sclerotia community in parentheses): *Virgisporangium* (0.417%), *Stenotrophobacter* (0.159%), *Skermanella* (1.234%), *Mesorhizobium* (0.086%), *Massilia* (0.255%), *Marmoricola* (0.594%), and *Dactylosporangium* (0.106%). Of these seven genera, three were enriched in Bottom Farm communities as a whole: *Virgisporangium*, *Skermanella*, and *Marmoricola*. At the Bottom Farm, two fungal genera met these criteria: *Paecilomyces* (0.825%) and *Colletotrichum* (0.151%), both of which were enriched in Bottom Farm communities as a whole. At the Stiles Farm, one bacterial genus met the aforementioned criteria – *Stenotrophobacter* (0.151%). Also at the Stiles Farm, three fungal genera met the criteria: *Preussia* (0.619%), *Hyponectriacea* species (0.873%), and *Alternaria* (12.8%). Two of those fungal genera, *Hyponectriacea* species and *Alternaria*, were enriched at the Stiles Farm as a whole.

The results of the βNTI analyses did not indicate deterministic selection for live sclerotia when compared to the other treatments for bacterial and fungal communities at both locations. At the Bottom Farm, the average βNTI values for bacterial communities were -5.18, -5.04, and -5.15 for live sclerotia, heat-killed sclerotia, and bulk soil, respectively. For fungal communities at the Bottom Farm, the average βNTI values were -1.44, -1.44, and -1.29 for live sclerotia, heat-killed sclerotia, and bulk soil, respectively. At the Stiles Farm, the average βNTI values for bacterial communities were -4.88, -4.72, and -5.05 for live sclerotia, heat-killed sclerotia, and bulk soil, respectively. For fungal communities at the Stiles Farm, the average βNTI values were -1.71, -1.5, and -1.59 for live sclerotia, heat-killed sclerotia, and bulk soil, respectively. The βNTI values at both locations for bacterial communities indicated that there was more phylogenetically similarity in those communities than what is expected under stochastic assembly models. On the other hand, the βNTI values at both locations for fungal communities indicated stochastic selection.

### 3.2 Comparison of communities between locations: 2024 cotton-growing season

**Figure 4:**
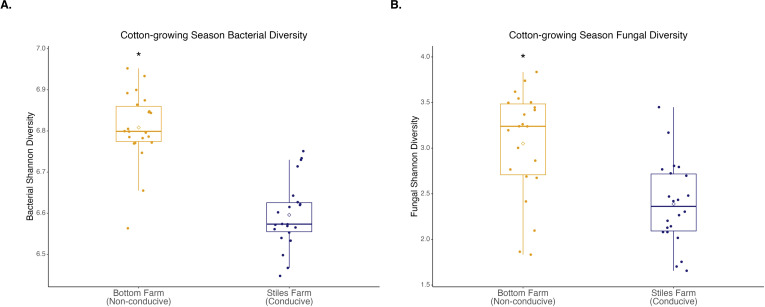
Microbial diversity during the 2024 cotton-growing season. Bacterial **(A)** and fungal **(B)** Shannon diversity was significantly (p<0.001) higher at the Bottom Farm. “*” represents a significant difference.

**Figure 5:**
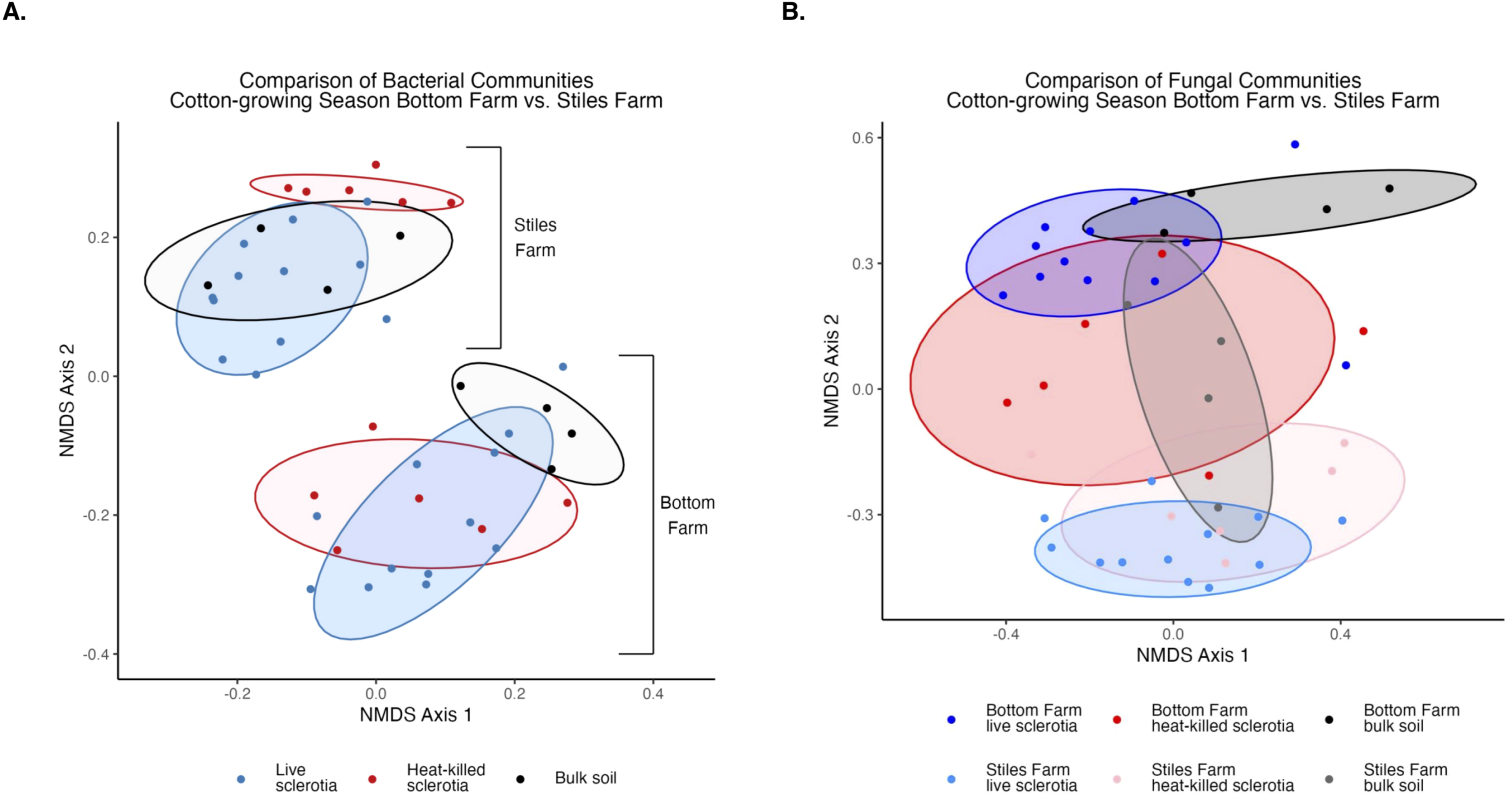
Comparison of microbial communities between locations during the 2024 cotton-growing season separated by sclerotia treatment. Bacterial **(A)** and fungal **(B)** communities were significantly (p<0.001) different by location.

Bacterial and fungal diversity was significantly (p<0.001) higher at the Bottom Farm than the Stiles Farm (**Figure 4**). When comparing the diversity of communities between locations, both bacterial (Fs=13.2, p<0.001) and fungal (Fs=6.7, p<0.001) communities significantly differed from one another (**Figure 5**). When looking within a location, sclerotia and bulk soil treatments significantly differed from one another. In bacterial communities at the Bottom Farm, the following treatments were significantly different from one another: live sclerotia – bulk soil (p=0.007) and heat-killed sclerotia – bulk soil (p=0.003). In sclerotia-associated bacterial communities at the Stiles Farm, only two treatments were significantly different from one another: live sclerotia – heat-killed sclerotia (p=0.003). In fungal communities at the Bottom Farm, the following treatments were significantly different from each other: live sclerotia – bulk soil (p=0.028), heat-killed sclerotia – bulk soil (p=0.031), and live sclerotia – heat-killed sclerotia (p=0.008). In fungal communities at the Stiles Farm, the following treatments were significantly different: live sclerotia – bulk soil (p<0.001), heat-killed – bulk soil (p=0.007), and live sclerotia – heat-killed sclerotia (p=0.015).

**Figure 6:**
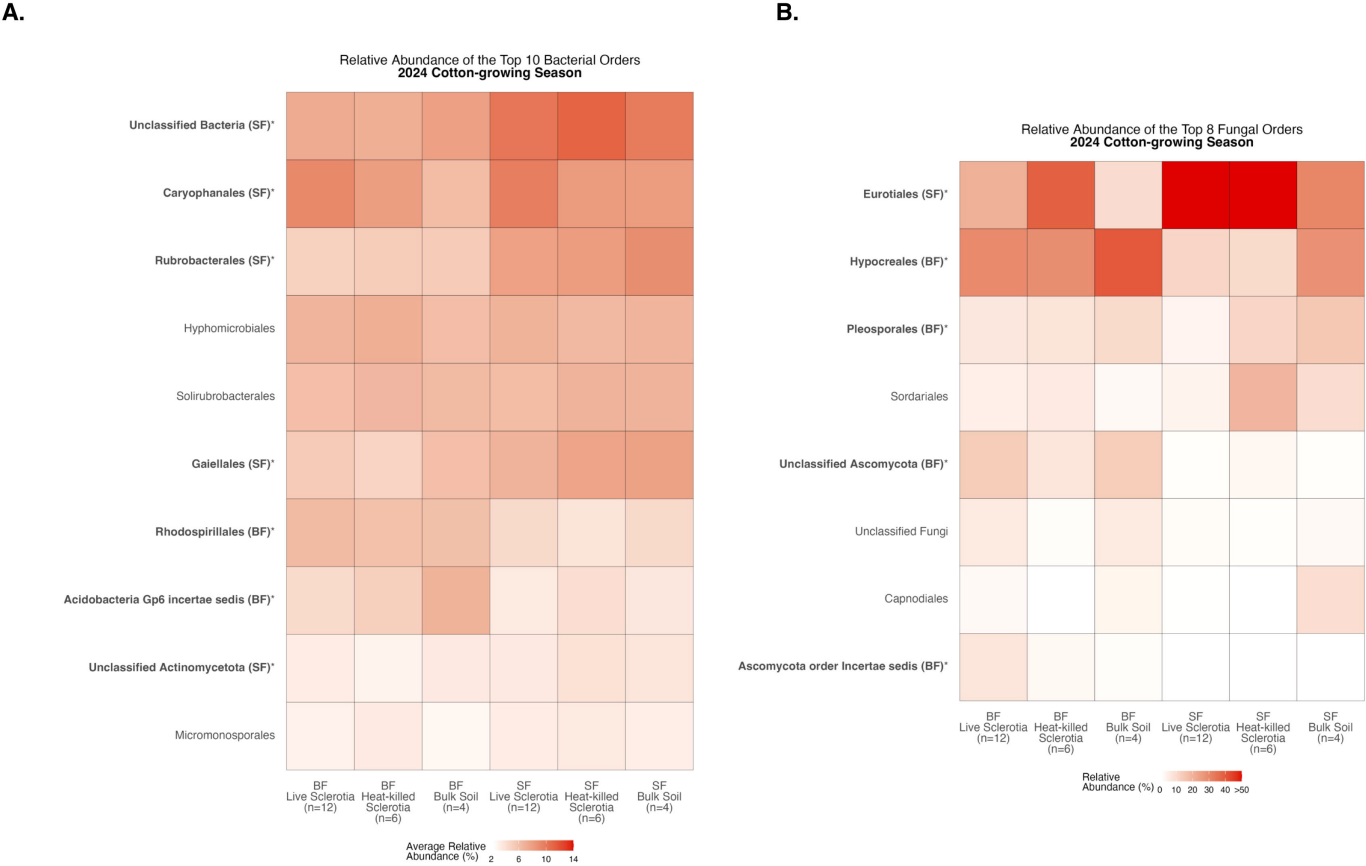
Heatmaps displaying diversity within location during the 2024 cotton-growing season. Bolded orders are significantly (p<0.05) different between the Bottom Farm (BF) and Stiles Farm (SF). “(BF)” or “(SF)” after a bolded order indicates which location had a significantly higher relative abundance of that order. **(A)** Comparison of bacterial communities. **(B)** Comparison of fungal communities.

Comparing relative abundance within a location, there were significant differences among the 10 most abundant bacterial orders averaged across samples in each of the sclerotia treatments and bulk soil (**Supplementary Table S1**). At the Bottom Farm, bacteria in the orders *Rhodospirillales* and Acidobacteria Gp6 incertae sedis were found at a significantly higher relative abundance than at the Stiles Farm (**Figure 6A**). At the Stiles Farm, bacteria in the orders Unclassified Bacteria, *Caryophanales*, *Rubrobacterales*, *Gaiellales*, and Unclassified Actinomycetota were found at a significantly higher relative abundance than at the Bottom Farm (**Figure 6A**). With fungal communities, there were significant differences among the eight most abundant fungal orders averaged across samples in each of the sclerotia treatments and bulk soil (**Supplementary Table S2**). At the Bottom Farm, fungi in the orders *Hypocreales*, *Pleosporales*, Unclassified Ascomycota, and Ascomycota order Incertae sedis were found at a significantly higher relative abundance than at the Stiles Farm (**Figure 6B**). At the Stiles Farm, fungi in only one order were found at a significantly higher relative abundance than at the Bottom Farm – *Eurotiales* (**Figure 6B**). The full breadth of bacterial and fungal alpha diversity during the 2024 cotton-growing season, at the order level, is shown in **Supplementary Figures S7**, **S8**, respectively.

In the Bottom Farm communities, the genus *Pseudomonas* made up 0.12% of the community, while members of the class Actinobacteria made up 17.9%. In the Stiles Farm communities, the genus *Pseudomonas* made up 0.02% of the community, while members of the class Actinobacteria made up 15.44%.

Based on the results of three LEfSe analyses, genera that were enriched in live sclerotia treatments when compared to bulk soil, but not enriched in heat-killed sclerotia, were selected. At the Bottom Farm, there were three bacterial genera that met these criteria (with the percentage that genus represents in the live sclerotia community in parentheses): *Robertmurraya* (0.275%), Gp10 (0.373%), and *Cellulomonas* (0.159%). Of these three genera, one was enriched in Bottom Farm communities as a whole – *Cellulomonas*. At the Bottom Farm, three fungal genera met these criteria: *Paecilomyces* (4.06%), *Mycoleptodiscus* (4.833%), and *Coniocessia* (0.043%). Of these three genera, two were enriched in Bottom Farm communities as a whole: *Paecilomyces* and *Mycoleptodiscus*. At the Stiles Farm, two bacterial genera met the aforementioned criteria: *Metabacillus* (0.28%) and *Domibacillus* (0.651%), both of which were enriched at the Stiles Farm as a whole. Also at the Stiles Farm, one fungal genus met the criteria – *Coniocessia* (0.038%).

The results of the βNTI analyses did not indicate deterministic selection for live sclerotia when compared to the other treatments for bacterial and fungal communities at both locations. At the Bottom Farm, the average βNTI values for bacterial communities were -4.49, -4.52, and -4.91 for live sclerotia, heat-killed sclerotia, and bulk soil, respectively. For fungal communities at the Bottom Farm, the average βNTI values were -1.31, -1.36, and -1.13 for live sclerotia, heat-killed sclerotia, and bulk soil, respectively. At the Stiles Farm, the average βNTI values for bacterial communities were -4.49, -4.39, and -4.51 for live sclerotia, heat-killed sclerotia, and bulk soil, respectively. For fungal communities at the Stiles Farm, the average βNTI values were -1.03, -1.02, and -1.39 for live sclerotia, heat-killed sclerotia, and bulk soil, respectively. The βNTI values at both locations for bacterial communities indicated that there was more phylogenetically similarity in those communities than what is expected under stochastic assembly models. On the other hand, the βNTI values at both locations for fungal communities indicated stochastic selection.

### 3.3 Comparison of communities between seasons: off versus cotton-growing season

**Figure 7:**
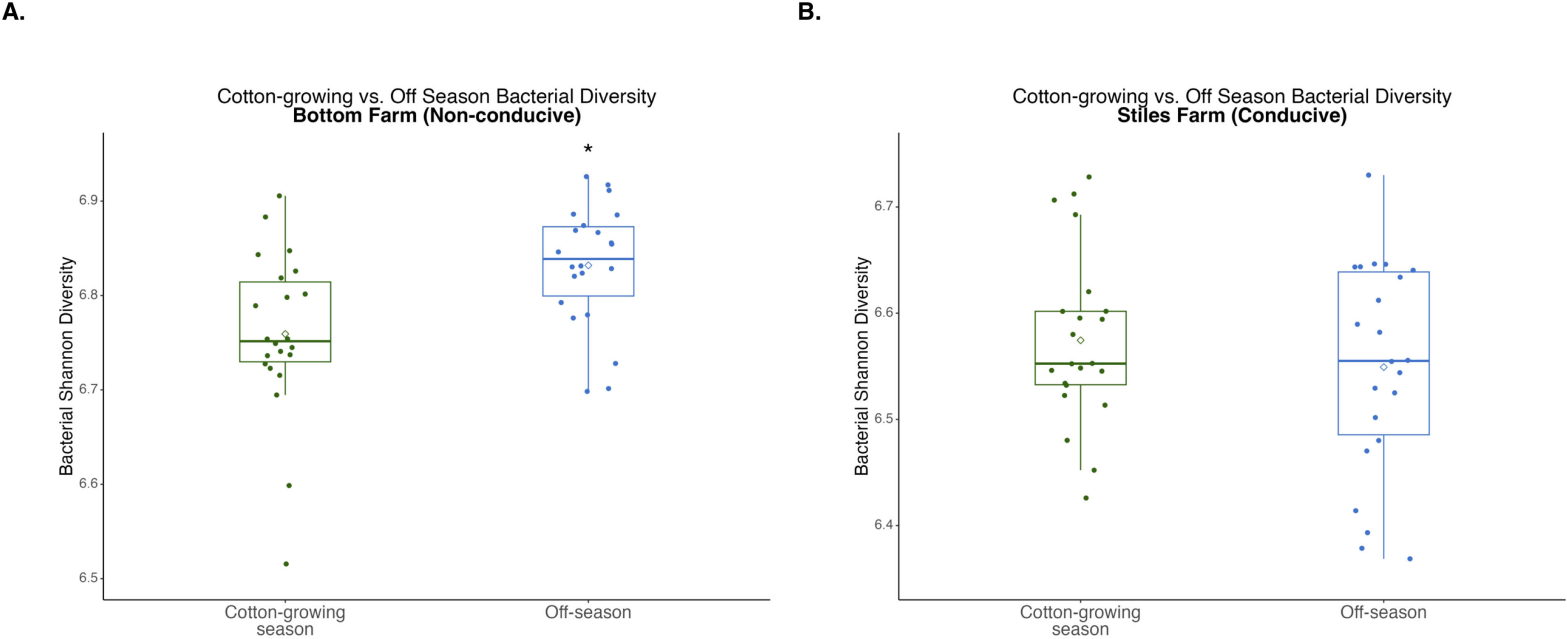
Comparison of bacterial diversity at the Bottom Farm **(A)** and Stiles Farm **(B)** between the off-season and 2024 cotton-growing season. Off-season bacterial Shannon diversity was significantly (p<0.001) higher at the Bottom Farm. “*” represents a significant difference.

**Figure 8:**
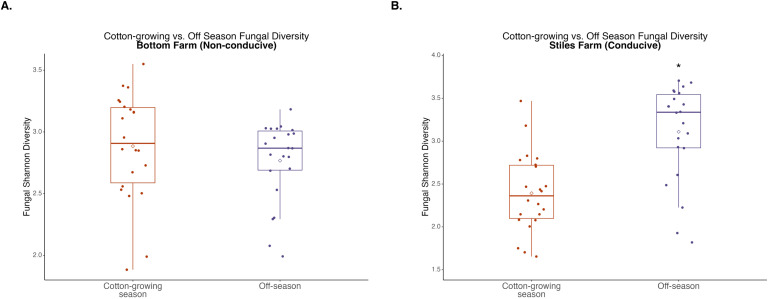
Comparison of fungal diversity at the Bottom Farm **(A)** and Stiles Farm **(B)** between the off-season and 2024 cotton-growing season. Off-season fungal Shannon diversity was significantly (p<0.001) higher at the Stiles Farm. “*” represents a significant difference.

When bacterial and fungal diversity during the off-season and cotton-growing seasons was compared at both locations, two significant differences were observed. Off-season bacterial diversity at the Bottom Farm was significantly (p<0.001) higher than the cotton-growing season at that location (**Figure 7**), and off-season fungal diversity at the Stiles Farm was significantly (p<0.001) higher than the cotton-growing season at that location (**Figure 8**). When comparing the diversity of the communities between seasons as a whole, or beta-diversity, both bacterial and fungal communities significantly (p<0.001) differed from one another. Between the cotton-growing and off seasons, sclerotia and bulk soil treatments differed significantly. In bacterial communities at the Bottom Farm, live sclerotia (p<0.001), heat-killed sclerotia (p=0.003), and bulk soil (p=0.023) were all significantly different between the seasons. In bacterial communities at the Stiles Farm, live sclerotia (p<0.001) and heat-killed sclerotia (p<0.001) were significantly different, but not the bulk soil communities. In fungal communities at the Bottom Farm, live sclerotia (p<0.001), heat-killed sclerotia (p=0.004), and bulk soil (p=0.023) were all significantly different by season. In fungal communities at the Stiles Farm, live sclerotia (p<0.001) were significantly different by season, however, heat-killed sclerotia and bulk soil were not.

**Figure 9:**
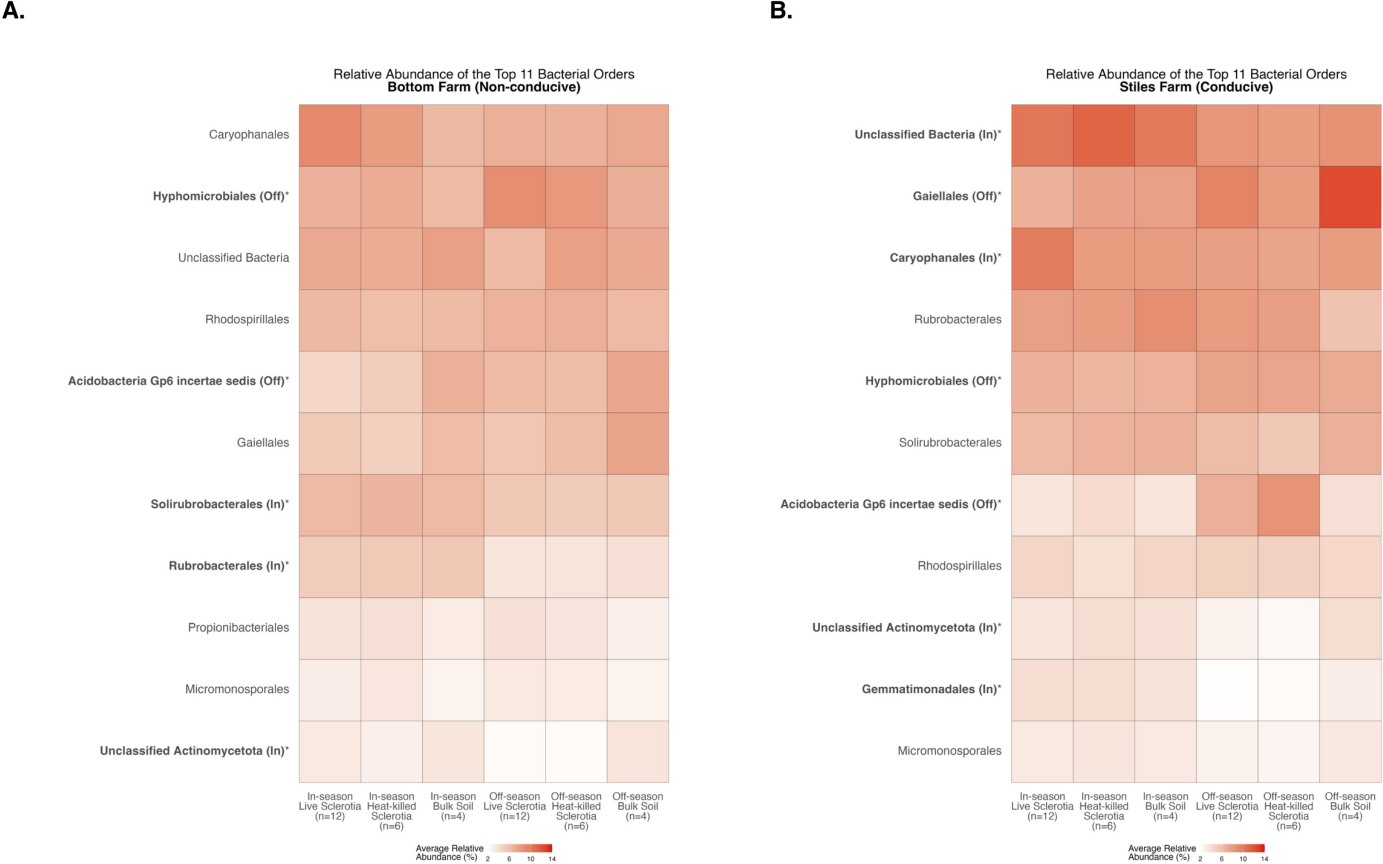
Heatmaps displaying diversity within location and season. Bolded orders are significantly (p<0.05) different between the cotton-growing and off season. “(Off)” or “(In)” after a bolded order indicates which season had a significantly higher relative abundance of that order. Comparison of bacterial communities at the Bottom Farm **(A)** and Stiles Farm **(B)**.

There were significant differences between the cotton-growing and off season among the 11 most abundant bacterial orders averaged across samples in each of the sclerotia treatments and bulk soil (**Supplementary Table S1**). At the Bottom Farm, bacteria in the orders *Hyphomicrobiales* and Acidobacteria Gp6 incertae sedis had a significantly higher relative abundance during the off-season than in the cotton-growing season (**Figure 9A**). On the other hand, bacteria in the orders *Solirubrobacterales*, *Rubrobacterales*, and Unclassified Actinomycetota had a significantly higher relative abundance during the cotton-growing season than in the off-season at the Bottom Farm (**Figure 9A**). At the Stiles Farm, bacteria in the orders *Gaiellales*, *Hyphomicrobiales*, and Acidobacteria Gp6 incertae sedis were found in a significantly higher relative abundance in the off-season than in the cotton-growing season (**Figure 9B**). Conversely, at the Stiles Farm, bacteria in the orders Unclassified Bacteria, *Caryophanales*, Unclassified Actinomycetota, and *Gemmatimonadales* had a significantly higher relative abundance during the cotton-growing season than the off-season (**Figure 9B**).

**Figure 10:**
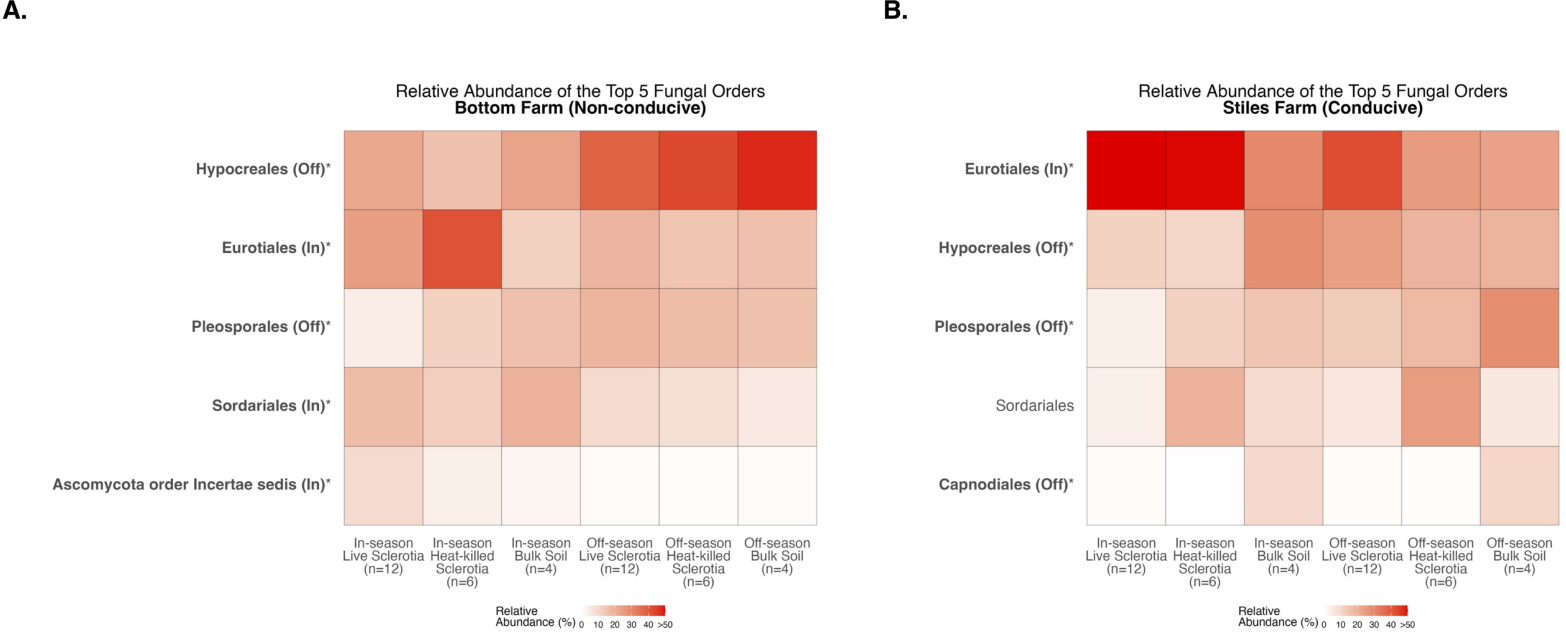
Heatmaps displaying diversity within location and season. Bolded orders are significantly (p<0.05) different between the cotton-growing and off season. “(Off)” or “(In)” after a bolded order indicates which season had a significantly higher relative abundance of that order. Comparison of fungal communities at the Bottom Farm **(A)** and Stiles Farm **(B)**.

With fungal communities, there were significant differences between the cotton-growing and off season among the five most abundant fungal orders averaged across samples in each of the sclerotia treatments and bulk soil (**Supplementary Table S2**). At the Bottom Farm, fungi in the orders *Hypocreales* and *Pleosporales* had a significantly higher relative abundance during the off-season when compared to the cotton-growing season (**Figure 10A**). Also at the Bottom Farm, fungi in the orders *Eurotiales*, *Sordariales*, and Ascomycota order Incertae sedis had a significantly higher relative abundance during the cotton-growing season when compared to the off-season (**Figure 10A**). At the Stiles Farm, fungi in the orders *Hypocreales*, *Pleosporales*, and *Capnodiales* had a significantly higher relative abundance during the off-season when compared to the cotton-growing season (**Figure 10B**). Conversely, at the Stiles Farm, fungi in the order *Eurotiales* were found in a significantly higher relative abundance during the cotton-growing season when compared to the off-season (**Figure 10B**). The full breadth of alpha diversity compared between the 2023–2024 off-season and 2024 cotton-growing season, at the order level, is shown in **Supplementary Figure S9** for bacterial communities and **Supplementary Figure S10** for fungal communities.

Taxa exclusively enriched in live sclerotia communities (both in the growing season and off-season) and not enriched in bulk soil communities were identified, based on the LEfSe analyses. At the Bottom Farm, five genera met these criteria: *Virgisporangium*, *Rubrobacter*, *Robertmurraya*, *Neobacillus*, and *Arboricoccus*. At the Stiles Farm, 10 genera met the criteria: *Oxalophagus*, *Oscillochloris*, *Neobacillus*, *Microlunatus*, *Metabacillus*, Gp4, *Ectobacillus*, *Archangium*, *Arboricoccus*, and *Amycolatopsis*. For fungal communities, no genera met these criteria at either location.

### 3.4 Comparison of communities over three seasons at the Stiles Farm

Evaluation of bacterial and fungal communities at the Stiles Farm for approximately nine months facilitated observation of changes in *P. omnivora* sclerotia-associated communities over time. A microbial order was considered differentially abundant between the cotton-growing seasons and the off-season if average relative abundance decreased from the 2023 cotton-growing season to the off-season, then increased from the off-season to the 2024 cotton-growing season, or vice versa. Change over time in the top 10 most abundant bacterial orders, associated with live sclerotia, can be seen in **Supplementary Table S3**. Of these 10 orders, two were differentially abundant between the cotton-growing seasons and the off-season: Acidobacteria Gp6 incertae sedis and *Gaiellales*. For both orders, average relative abundance increased from the 2023 cotton-growing season to the off-season, then decreased from the off-season to the 2024 cotton-growing season. In both cases, the average relative abundance of bacteria in the orders was significantly higher in the off-season than in both cotton-growing seasons. Change over time in the top 10 most abundant bacterial orders, associated with heat-killed sclerotia, can be seen in **Supplementary Table S3**. Of these 10 orders, five were differentially abundant between the cotton-growing seasons and the off-season: *Acidimicrobiales*, Acidobacteria Gp6 incertae sedis, *Gaiellales*, *Micromonosporales*, and *Rubrobacterales*. In bacteria classified in *Acidimicrobiales*, *Micromonosporales*, and *Rubrobacterales*, the average relative abundance decreased from the 2023 cotton-growing season to the off-season, then increased from the off-season to the 2024 cotton-growing season. In bacteria classified in Acidobacteria Gp6 incertae sedis and *Gaiellales*, average relative abundance increased from the 2023 cotton-growing season to the off-season, then decreased from the off-season to the 2024 cotton-growing season. Change over time in the top 10 most abundant bacterial orders, associated with bulk soil, can be seen in **Supplementary Table S3**. Of these 10 orders, seven were differentially abundant between the cotton-growing seasons and the off-season: *Acidimicrobiales*, *Caryophanales*, *Gaiellales*, *Micromonosporales*, *Rhodospirillales*, *Rubrobacterales*, and *Solirubrobacterales*. In bacteria classified in *Acidimicrobiales*, *Rhodospirillales*, and *Rubrobacterales*, average relative abundance decreased from the 2023 cotton-growing season to the off-season, then increased from the off-season to the 2024 cotton-growing season. In bacteria classified in *Caryophanales*, *Gaiellales*, *Micromonosporales*, and *Solirubrobacterales*, average relative abundance increased from the 2023 cotton-growing season to the off-season, then decreased from the off-season to the 2024 cotton-growing season. The differences in the relative abundance of bacterial communities associated with bulk soil were numerically different over time, but not statistically.

Change over time in the top six most abundant fungal orders, associated with live sclerotia, can be seen in **Supplementary Table S4**. Of the six orders, only one was differentially abundant between the cotton-growing seasons and the off-season: *Sordariales*. Fungi in the order *Sordariales* increased in average relative abundance from the 2023 cotton-growing season to the off-season, then decreased from the off-season to the 2024 cotton-growing season, but the change was not significantly different. Change over time in the top six most abundant fungal orders, associated with heat-killed sclerotia, can be seen in **Supplementary Table S4**. Of the six orders, two were differentially abundant between the cotton-growing seasons and the off-season: *Hypocreales* and *Sordariales*. Fungi in both of these orders increased from the 2023 cotton-growing season to the off-season, and then decreased from the off-season to the 2024 cotton-growing season, but these changes were not statistically significant. Change over time in the top six most abundant fungal orders, associated with bulk soil, can be seen in **Supplementary Table S4**. Of the six orders, three were differentially abundant between the cotton-growing seasons and the off-season: *Capnodiales*, *Hypocreales*, and *Pleosporales*. Fungi in *Capnodiales* and *Pleosporales* increased from the 2023 cotton-growing season to the off-season, then decreased from the off-season to the 2024 cotton-growing season. Conversely, fungi in the order Hypocreales decreased from the 2023 cotton-growing season to the off-season, then increased from the off-season to the 2024 cotton-growing season. These differences in relative abundance over time were not statistically significant.

## 4 Discussion

This study provides the first comprehensive look at *P. omnivora* sclerotia-associated microorganisms in soils with contrasting histories of support for CRR, as well as documents how these communities change over time. The findings demonstrate that microbial communities differ in both diversity and composition across locations, seasons, and treatments. Specifically, microbial diversity was consistently higher at the Bottom Farm compared to the Stiles Farm. This was particularly evident in bacterial communities during the off-season and in both bacterial and fungal communities during the 2024 cotton-growing season. Given the absence of CRR at the Bottom Farm, this observation aligns with previous findings that associate increased microbial diversity with reduced CRR incidence (Chavez et al., 1976; Streets and Bloss, 1973). These findings suggest that part of the Bottom Farm’s lack of CRR could be due to its microbial diversity. This hypothesis could be tested by incorporating green manure at varying rates into a CRR-infested field, followed by observing CRR progression in that field subsequently planted with cotton, as was done by Chavez et al. (1976), and then evaluating soil microflora using metabarcoding. Such an experiment could clarify the link between microbial diversity and CRR incidence, providing non-microbial soil properties are not substantially changed by treatments. This study provides insight beyond community diversity metrics that show how microbial communities associated with *P. omnivora* sclerotia are qualitatively different between location and season, as well as quantitatively different when it comes to the relative abundance of microbial taxa found in both locations and seasons.

A core hypothesis of this study was that live sclerotia of *P. omnivora* selectively recruit particular microbial taxa. To investigate this, we used LEfSe analysis to identify microbial biomarkers enriched in live sclerotia relative to bulk soil and heat-killed sclerotia, and used βNTI to assess the nature of the community assembly processes. The LEfSe results show several bacterial and fungal genera are significantly enriched in live sclerotia across both seasons and test locations. During the off-season at the CRR non-conducive Bottom Farm, OTUs that belong to the bacterial genera such as *Virgisporangium*, *Skermanella*, *Marmoricola*, and fungal genera like *Paecilomyces* and *Colletotrichum* were enriched in live sclerotia. However, at the conducive Stiles Farm, *Stenotrophobacter*, *Alternaria*, and *Preussia* were among the enriched taxa. Many of these genera, particularly *Paecilomyces*, are known producers of secondary metabolites including antibiotics, antifungals, and nematocidal compounds (Hirota et al., 1991; Dai et al., 2020; Shi et al., 2025). The presence of actinobacteria such as *Marmoricola* and *Virgisporangium* aligns with broader patterns seen in sclerotia-forming pathogens like *Sclerotinia sclerotiorum*, where actinobacteria often act as early colonizers or antagonists. In the 2024 cotton-growing season, live sclerotia continued to enrich distinct taxa. At the Bottom Farm, bacterial genera *Cellulomonas* and *Robertmurraya*, along with fungal genera *Paecilomyces* and *Mycoleptodiscus*, were dominant. At the Stiles Farm, Metabacillus, Domibacillus, and *Coniocessia* were enriched. Notably, many of these genera are culturable, facilitating future mechanistic studies.

During both the 2023–2024 off-season and the 2024 cotton-growing season, representation of the genus *Pseudomonas* and members of the class Actinobacteria was evaluated because of findings by Zuberer et al. (1988) in which fluorescent pseudomonads and actinomycetes were found in association with *P. omnivora* sclerotia. The present study also found those groups of bacteria in association with live *P. omnivora* sclerotia, but as a low percentage of the community. This illustrates the ability of sequencing methods to more comprehensively identify the makeup of microbial communities, as compared to methods relying on selective media alone.

At the Stiles Farm, the three-season comparison (2023 cotton-growing season, 2023–2024 off-season, 2024 cotton-growing season) noted a snapshot of temporal dynamics. Some bacterial orders, such as Acidobacteria Gp6 incertae sedis and *Gaiellales*, showed consistent seasonal shifts, increasing in the off-season and decreasing during the growing season. However, not all patterns were conserved across niches. For instance, *Rubrobacterales* OTUs increased in abundance in bulk soil and heat-killed sclerotia communities during cotton seasons, but not in live sclerotia communities. Such differences suggest that live sclerotia may represent a distinct ecological niche with unique seasonal dynamics. Fungal communities also shifted seasonally but less consistently across treatments. Orders such as *Sordariales* were enriched in off-season sclerotia treatments but not in bulk soil. In contrast, fungi in *Capnodiales* and *Pleosporales* were more abundant in off-season bulk soils but not in sclerotia. These observations further suggest that fungal community dynamics are more stochastic and niche-specific compared to bacterial counterparts.

βNTI metrics are more recent indices used to evaluate deterministic selection in microbiome communities. This test helps evaluate whether a community structure is random or has been guided by a specific recruitment strategy. Specifically, it helps compare the observed phylogenetic dissimilarity between communities to a null distribution generated through randomizations, thereby allowing inference about the relative influence of deterministic versus stochastic assembly mechanisms. A βNTI value greater than +2.0 indicates that the phylogenetic composition of communities is more dissimilar than expected by chance, whereas value less than −2.0 reflects more phylogenetically similarity than expected under stochastic assembly models. Across all bacterial communities (live and heat-killed sclerotia and bulk soil), βNTI values were strongly negative ranging from -4.3 to -5.2, suggesting deterministic assembly driven by environmental filtering or host selection. In contrast, fungal βNTI values hovered between -1.0 and -1.7, indicating stochastic assembly dominated by random colonization or dispersal. While deterministic selection is expected in structured niches such as live and dead sclerotia, the observation of strongly negative βNTI values in bulk soil was unexpected. This pattern suggests that even bulk soil exerts a consistent selection pressure on bacterial communities. Such homogeneity may be driven by uniform soil physicochemical conditions, recurring plant-derived inputs (e.g., root exudates), or the residual effects of crop-associated organic matter. Although these factors likely contribute to the observed pattern, their specific roles remain unresolved within the scope of this study.

Microbial community comparisons between the 2023–2024 off-season and the 2024 cotton-growing season demonstrated the set of taxa that may be responsible for differences in CRR occurrence at the locations of interest. The comparison across communities allowed for the selection of genera that were enriched in live sclerotia, specifically in live sclerotia during the cotton-growing season. If the absence of CRR in some soils is microbially mediated, those microorganisms would be expected to be most active during the months when CRR manifests in the field. At the Bottom Farm, five bacterial genera met the enrichment criteria, including *Rubrobacter*, a member of the thermophilic order *Rubrobacterales*, that showed significantly higher relative abundance during the cotton-growing season. Given their thermotolerance (Albuquerque and da Costa, 2014), the higher relative abundance during the summer months is not unexpected. Although not associated with orders differing in relative abundance between the seasons, the selected genera *Virgisporangium* and *Neobacillus* may show functional relevance for competition with fungal pathogens. Characteristics of *Virgisporangium* are not well known, and the possibility of antibiotic production should be evaluated, as other members of the Actinomycetes produce such compounds. The genus *Neobacillus* was recently reclassified from the genus *Bacillus*, a genera noted for their secondary metabolic potential along with effects on plant growth (Kloepper et al., 2007), fungal modulation (Anckaert et al., 2024), and overall microbiome alterations (Stein, 2005; Adesemoye et al., 2025). *Neobacillus* was also enriched in live sclerotia communities during the cotton-growing season at the Stiles Farm, which is conducive to CRR. At the Stiles Farm, ten bacterial genera met the enrichment criteria, though none belonged to orders with seasonally differential abundance. Notably, all enriched bacterial genera from both locations are described in the literature as culturable.

The comparison of microbial communities at the Stiles Farm over three seasons allowed for the investigation of microorganisms that are differentially abundant between the cotton-growing and off seasons for each of the niches evaluated in this study. There were some bacterial orders that exhibited different seasonal shifts between the niches. Acidobacteria Gp6 incertae sedis was found at a higher relative abundance during the off-season for the sclerotia treatments, but this was not the case in bulk soil. Acidobacteria are well-known oligotrophs and perhaps their higher presence in off-season sclerotia is due to the low nutrient environment, outcompeting copiotrophs. However, *Acidimicrobiales* and *Rubrobacterales* as also oligotrophs but were found at a higher relative abundance during the cotton-growing seasons in the heat-killed sclerotia and bulk soil communities, but not the live sclerotia communities. *Micromonosporales* was found at a higher relative abundance during the cotton-growing seasons for heat-killed sclerotia communities, but was more abundant during the off-season in bulk soil. However, this could be due to their high stress-tolerance and, as mentioned earlier, might enable actinobacterial orders such as these to thrive. *Caryophanales* and *Solirubrobacterales* were found at a higher relative abundance during the off-season in bulk soil communities, but not in the sclerotia treatments. Among the aforementioned differences in seasonal shifts by niche, *Gaiellales* was the only order that was differentially abundant in the same way across the sclerotia treatments and bulk soil.

## 5 Conclusions

Overall, this study presents the first detailed characterization of microorganisms associated with *P. omnivora* sclerotia in different cotton-producing soils. Microbial diversity was higher at the non-conducive Bottom Farm, especially during the off-season and 2024 cotton-growing season, aligning with the hypothesis that greater microbial diversity contributes to disease suppression. LEfSe analyses revealed site- and season-specific microbial taxa enriched in live sclerotia. At the Bottom Farm, genera such as *Paecilomyces*, *Marmoricola*, and *Virgisporangium* were abundant. At the Stiles Farm, taxa like *Stenotrophobacter* and *Alternaria* dominated. These genera, including culturable ones like *Neobacillus* and *Metabacillus*, are known for antimicrobial potential. Further investigation of the culturable bacteria and fungi identified in this study should be evaluated *in vitro* for the ability to either suppress or encourage the growth of *P. omnivora*. βNTI values showed deterministic bacterial assembly across treatments, even in bulk soil, while fungal communities appeared stochastically assembled. This suggests a conditional selective recruitment where bacteria are selectively enriched on active sclerotia, while fungal recruitment is less structured. The seasonal shifts in specific bacterial orders (e.g., *Rubrobacterales*, Acidobacteria Gp6) and fungal taxa (e.g., *Sordariales*) highlighted niche- and time-dependent dynamics. These patterns parallel those observed in sclerotia communities of other pathogens like *Sclerotinia* and *Rhizoctonia*. Our findings support the view that *P. omnivora* sclerotia serve as ecological hubs, shaping microbial communities with possible implications for disease suppression. Several enriched taxa are culturable, offering candidates for future biocontrol studies.

## Data Availability

The datasets presented in this study can be found in online repositories. The names of the repository/repositories and accession number(s) can be found below: https://www.ncbi.nlm.nih.gov/, BioProject PRJNA1289165.
