# Supplementary material for: Identification of microbial communities associated with Phymatotrichopsis omnivora sclerotia in two Texas fields
Source: Front Microbiomes. 2025 Nov 28;4:1666691. doi: 10.3389/frmbi.2025.1666691 (PMC12993665; doi:10.3389/frmbi.2025.1666691)
Supplement: Supplementary file 1 [file DataSheet1.docx]

***Supplementary Material***

**1 Supplementary figures**

**B**

**A**

**
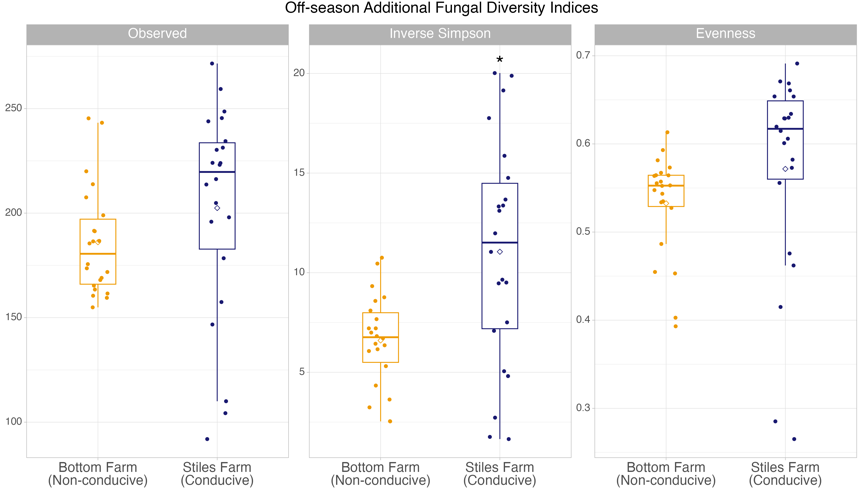

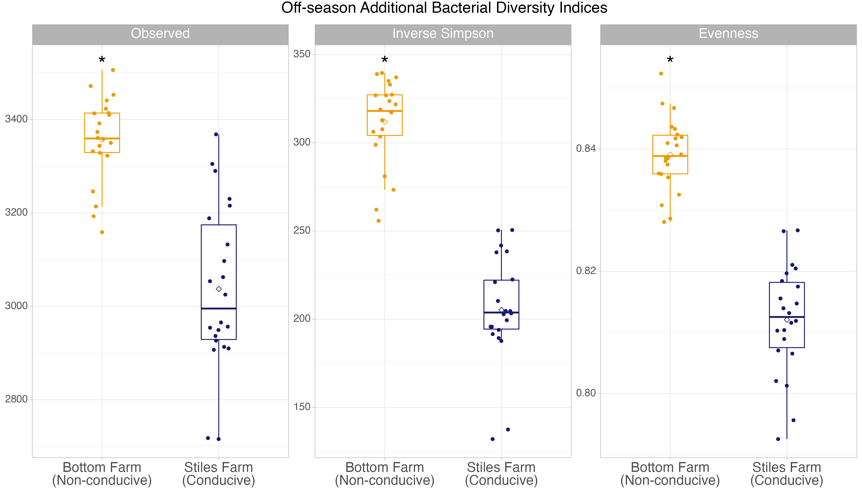
**

**Supplementary Figure 1.** Microbial diversity during the 2023-2024 off-season. **(A)** Bacterial observed and inverse Simpson diversity, as well as evenness, were significantly (p<0.001) higher at the Bottom Farm. **(B)** Fungal inverse Simpson diversity was significantly (p=0.002) higher at the Stiles Farm. “*” represents a significant difference.

**
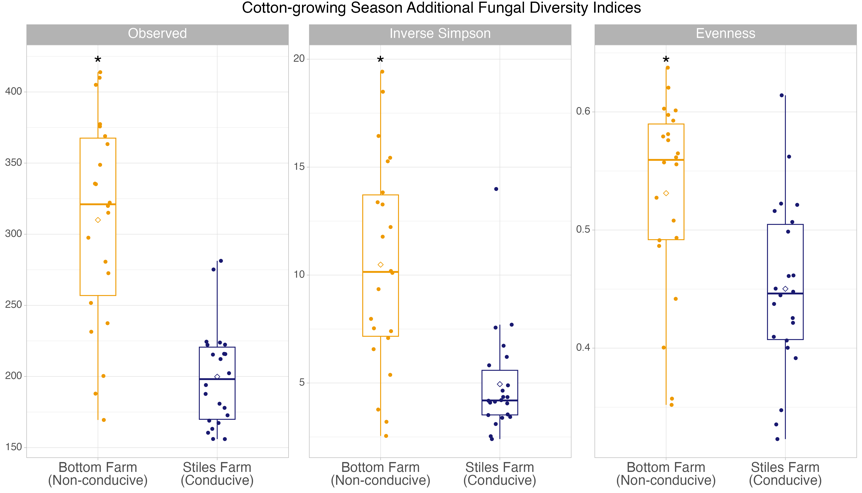

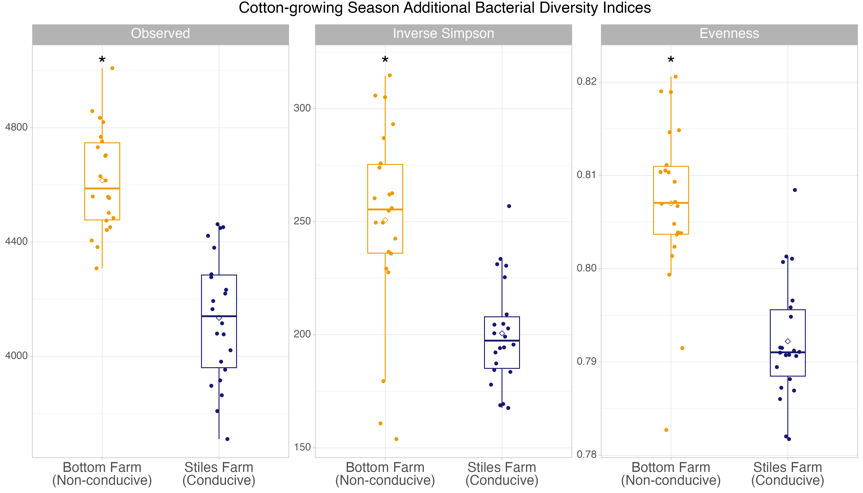
**

**B**

**A**

**Supplementary Figure 2.** Microbial diversity during the 2024 cotton-growing season. **(A)** Bacterial observed and inverse Simpson diversity, as well as evenness, were significantly (p<0.001) higher at the Bottom Farm. **(B)** Fungal observed (p<0.001) and inverse Simpson diversity (p<0.001), as well as evenness (p=0.001), were significantly higher at the Bottom Farm. “*” represents a significant difference.

**
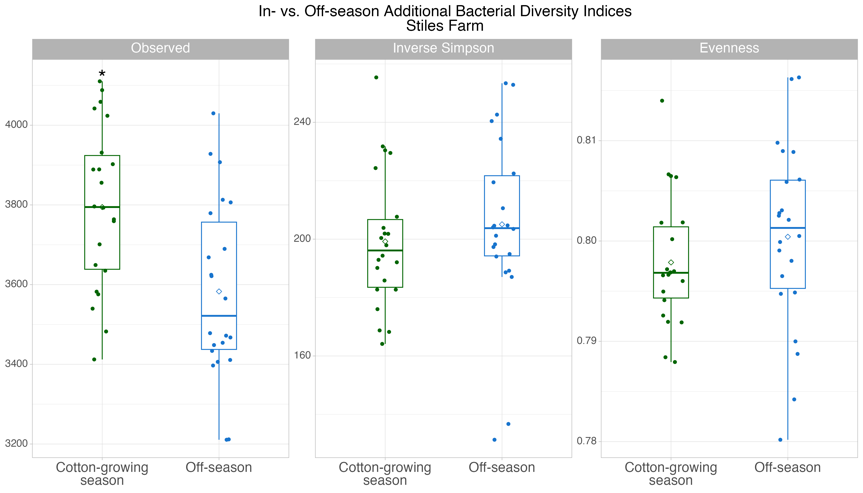

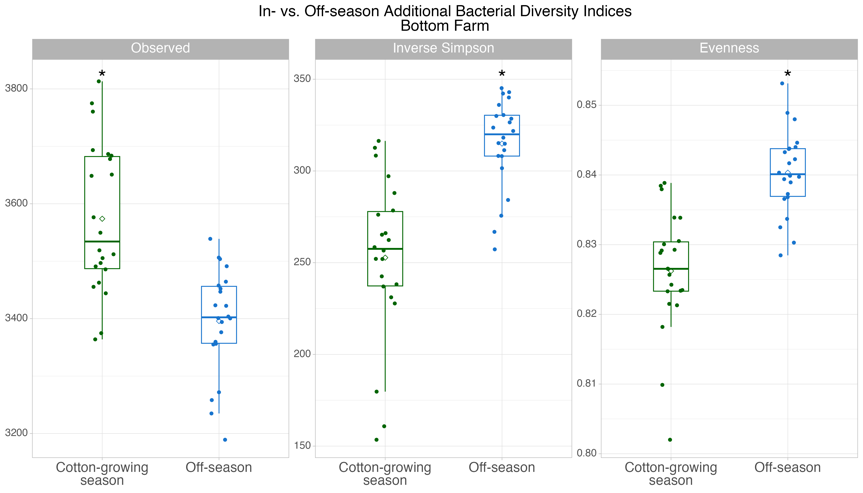
**

**A**

**B**

**Supplementary Figure 3.** Comparison of bacterial diversity at the Bottom Farm **(A)** and Stiles Farm **(B)** between the off-season and 2024 cotton-growing season. In-season bacterial observed diversity was significantly (p<0.001) higher at the Bottom Farm, while off-season inverse Simpson diversity and evenness were significantly (p<0.001) higher at the same location. In-season bacterial observed diversity was significantly (p=0.002) higher at the Stiles Farm. “*” represents a significant difference.

**A**

**B**


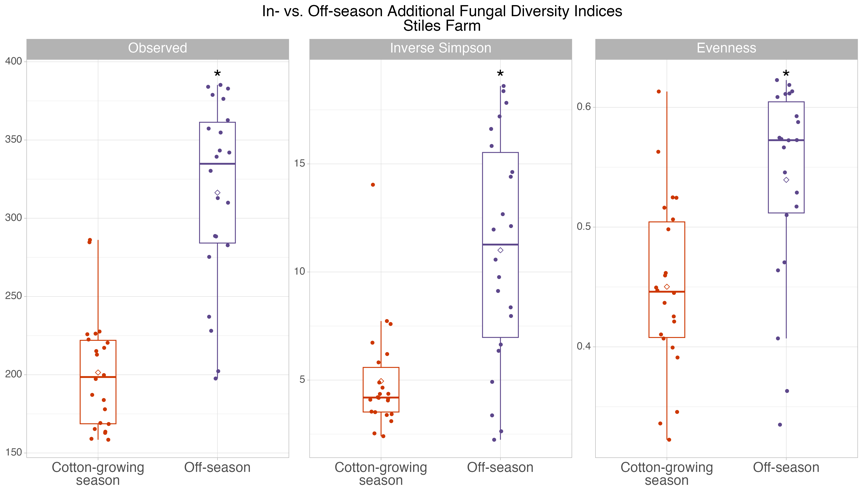

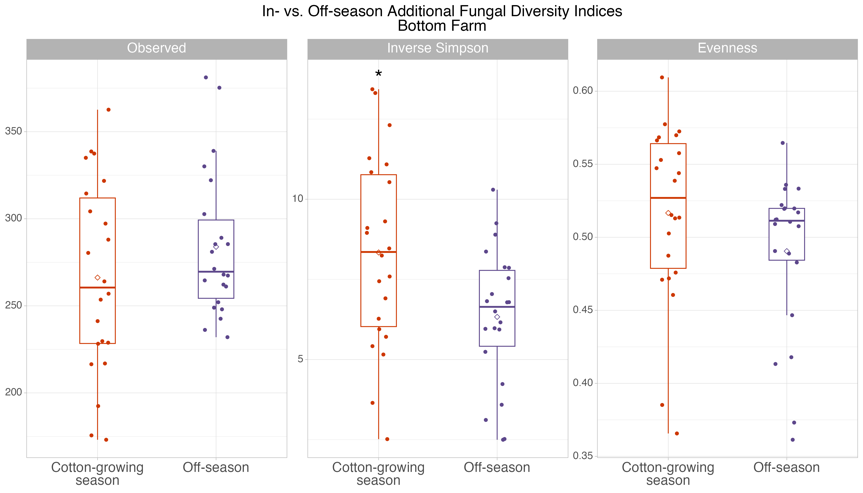


**Supplementary Figure 4.** Comparison of fungal diversity at the Bottom Farm **(A)** and Stiles Farm **(B)** between the off-season and 2024 cotton-growing season. Cotton-growing season fungal inverse Simpson diversity was significantly (p=0.014) higher at the Bottom Farm. Off-season observed and inverse Simpson diversity, as well as evenness, were significantly (p<0.001) higher at the Stiles Farm. “*” represents a significant difference.


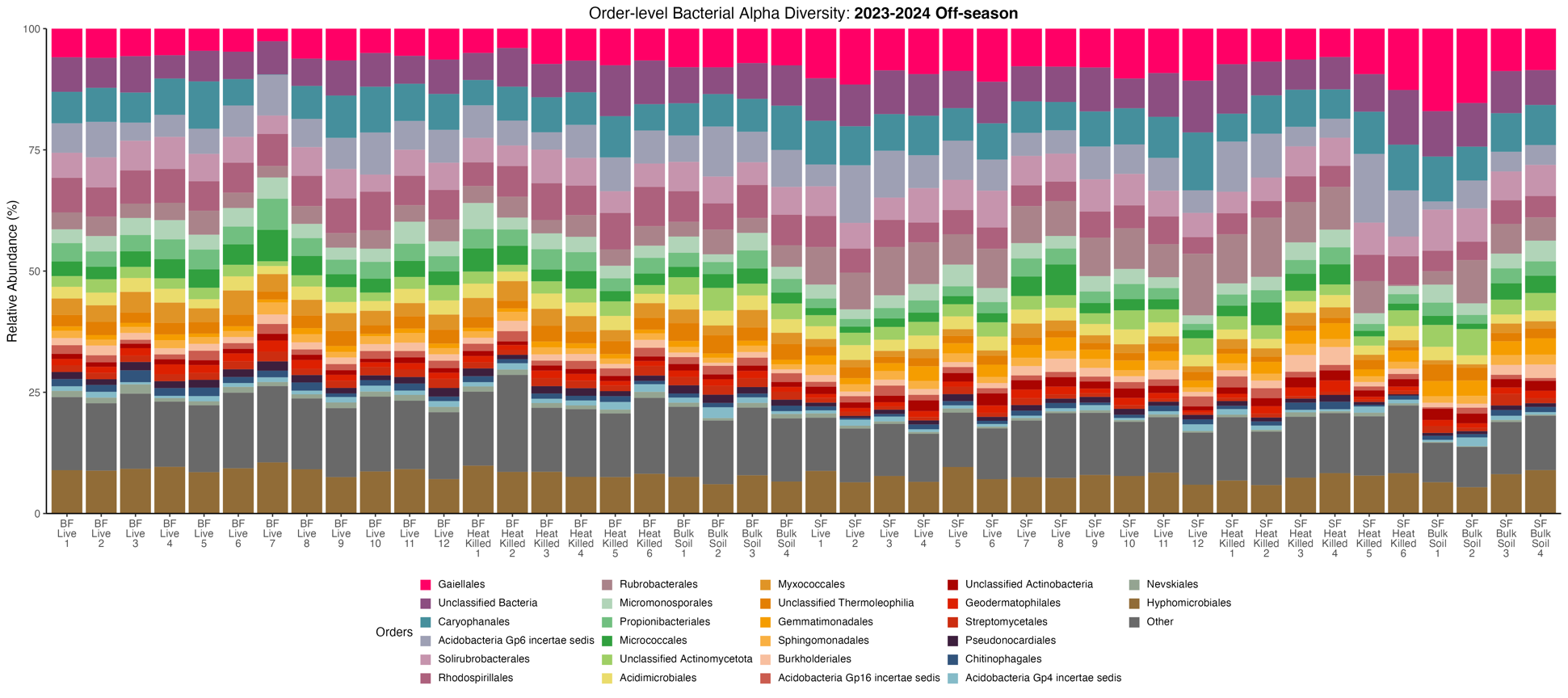


**Supplementary Figure 5.** Stacked bar chart showing the full breadth of bacterial diversity in all samples from the 2023-2024 off-season. Orders classified as “Other” were found at a relative abundance less than 2%. “BF” stands for Bottom Farm, and “SF” stands for Stiles Farm. “Live” represents live sclerotia samples, and “Heat killed” represents heat-killed sclerotia samples.


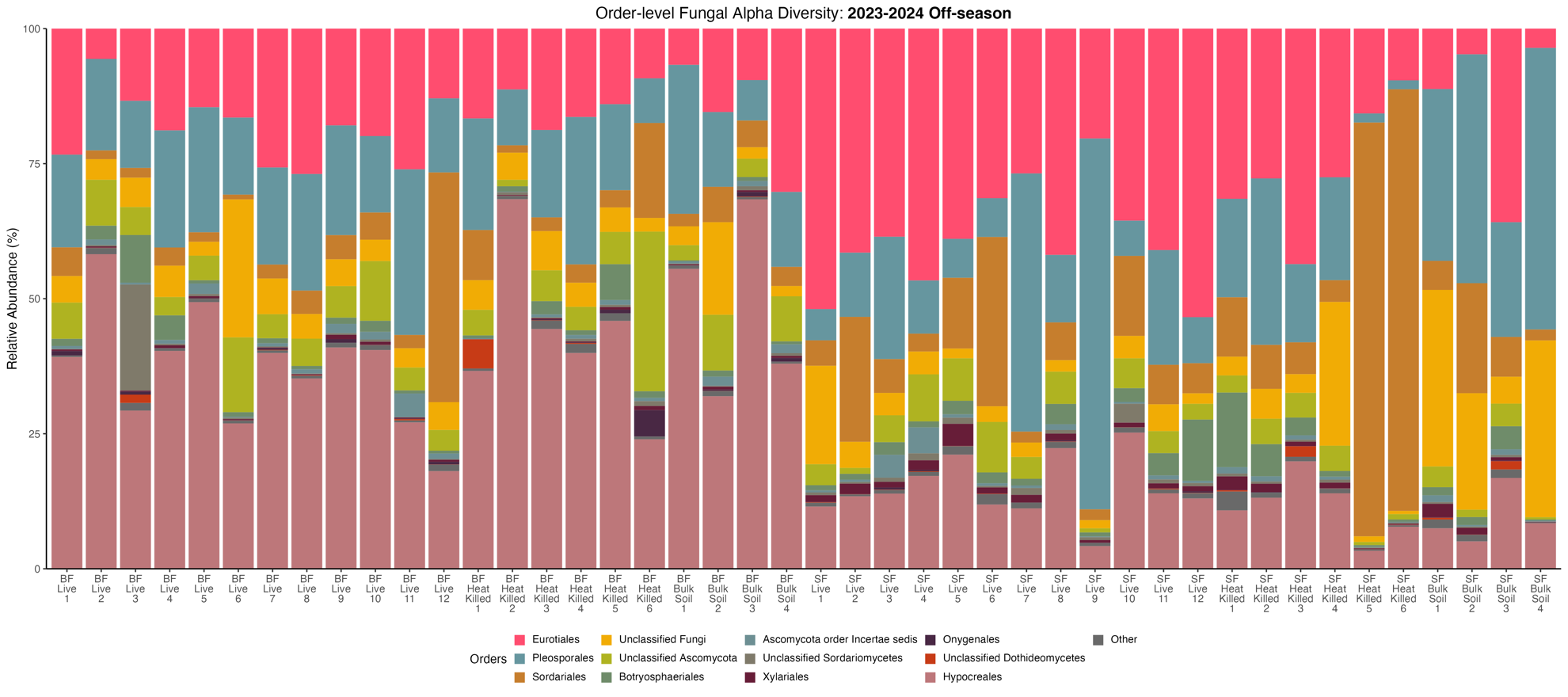


**Supplementary Figure 6.** Stacked bar chart showing the full breadth of fungal diversity in all samples from the 2023-2024 off-season. Orders classified as “Other” were found at a relative abundance less than 4%. “BF” stands for Bottom Farm, and “SF” stands for Stiles Farm. “Live” represents live sclerotia samples, and “Heat killed” represents heat-killed sclerotia samples.


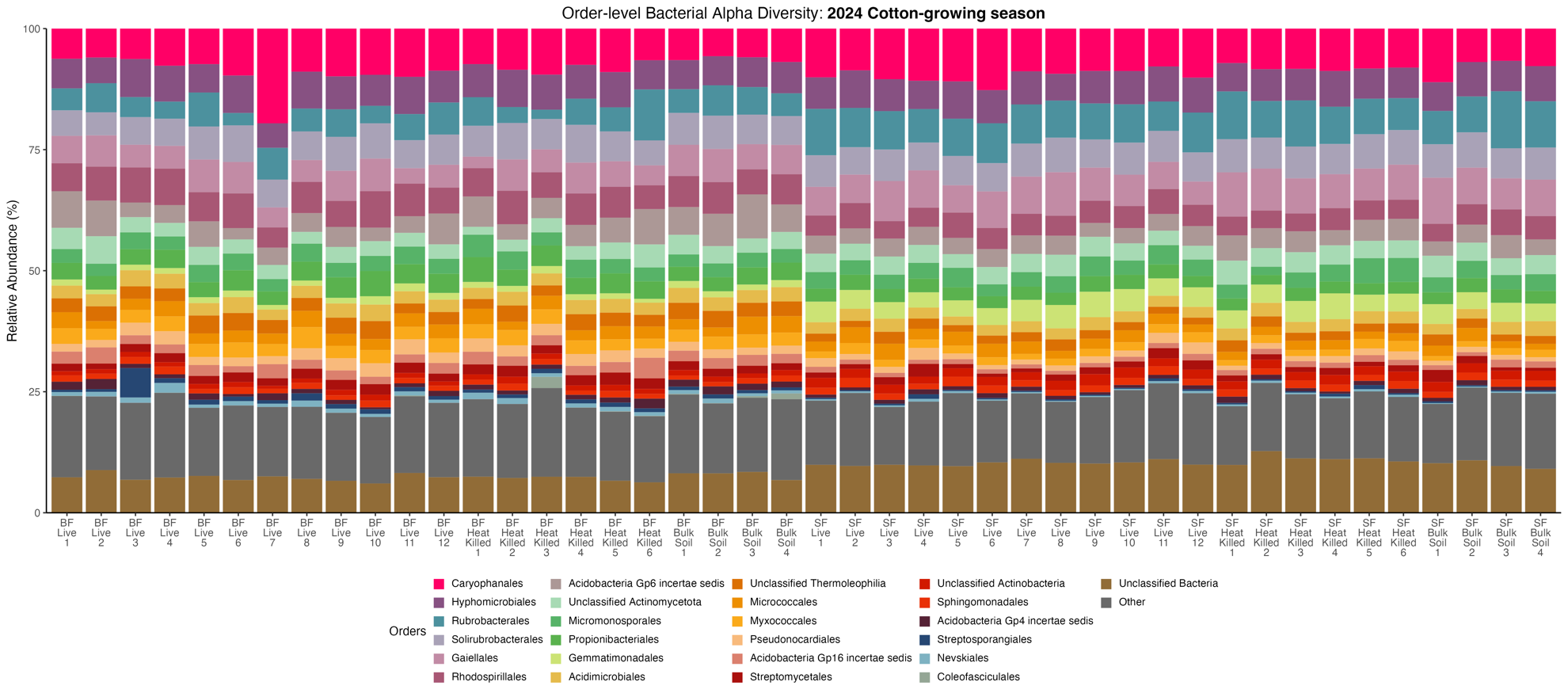


**Supplementary Figure 7.** Stacked bar chart showing the full breadth of bacterial diversity in all samples from the 2024 cotton-growing season. Orders classified as “Other” were found at a relative abundance less than 2%. “BF” stands for Bottom Farm, and “SF” stands for Stiles Farm. “Live” represents live sclerotia samples, and “Heat killed” represents heat-killed sclerotia samples.


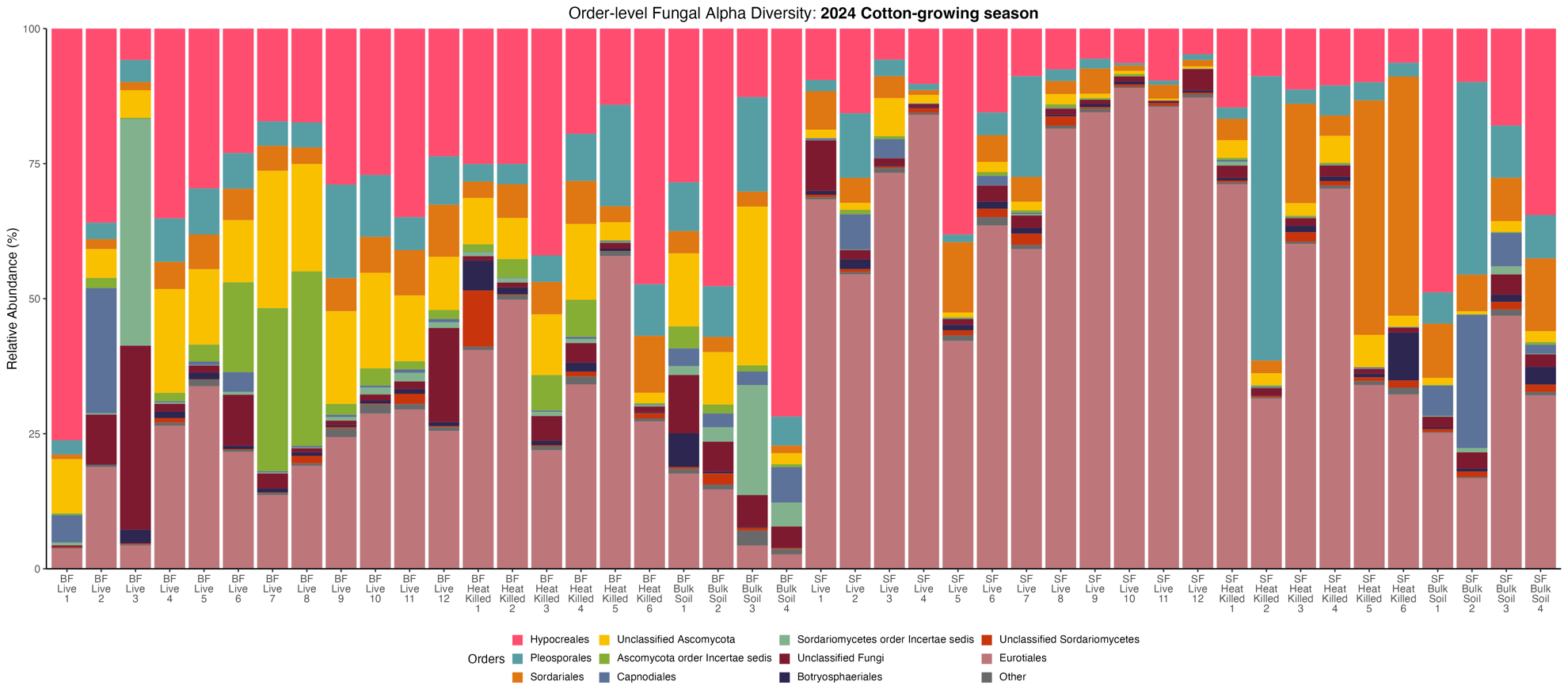


**Supplementary Figure 8.** Stacked bar chart showing the full breadth of fungal diversity in all samples from the 2024 cotton-growing season. Orders classified as “Other” were found at a relative abundance less than 3%. “BF” stands for Bottom Farm, and “SF” stands for Stiles Farm. “Live” represents live sclerotia samples, and “Heat killed” represents heat-killed sclerotia samples.


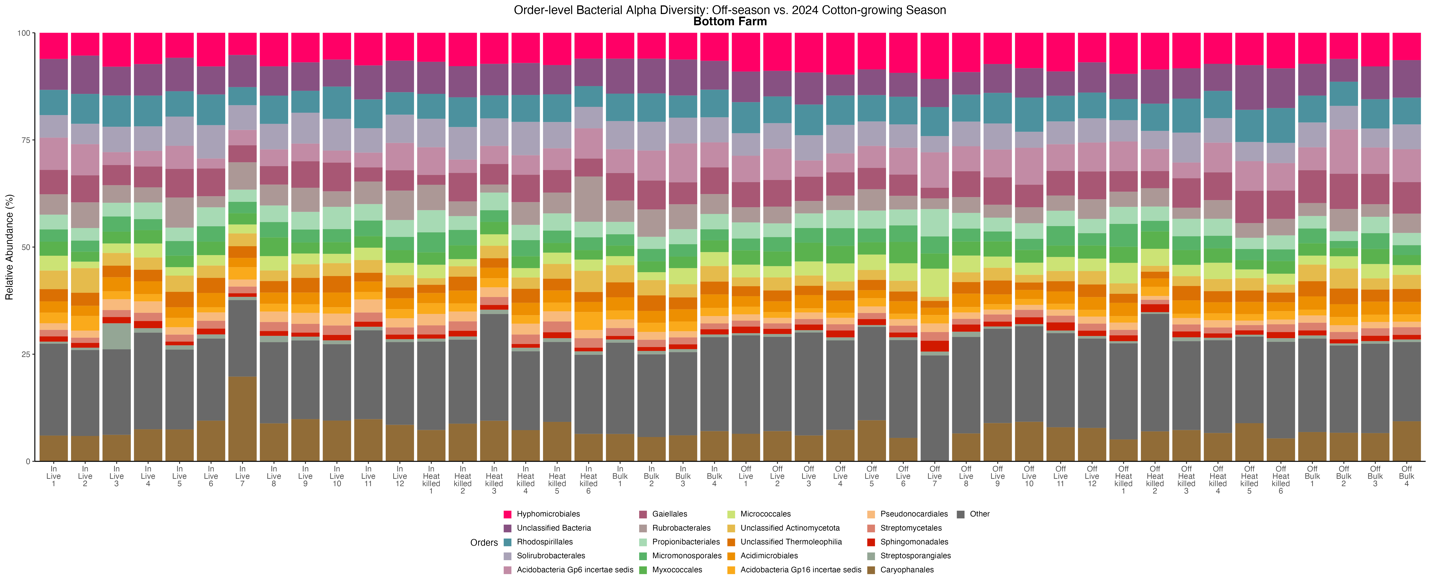


**A**

**B**


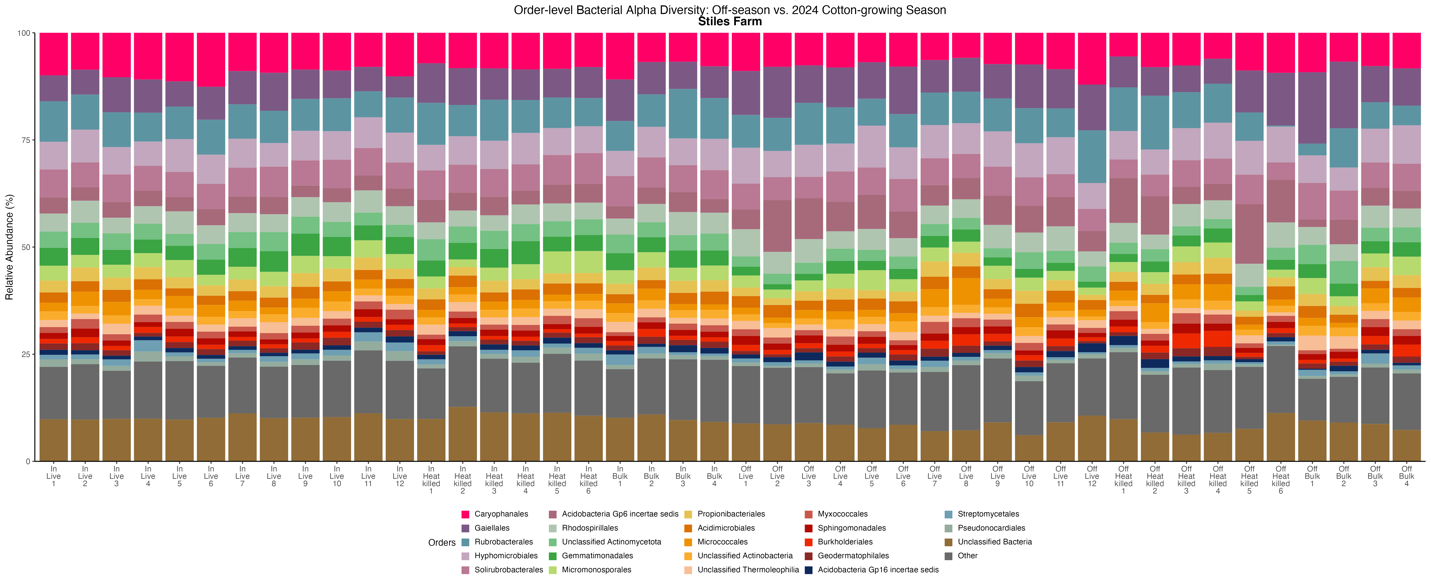


**Supplementary Figure 9.** Stacked bar charts showing bacterial diversity of all samples, at both locations, during the off-season and 2024 cotton-growing season. **(A)** Bottom Farm. Orders classified as “Other” were found at a relative abundance less than 2.5%. **(B)** Stiles Farm. Orders classified as “Other” were found at a relative abundance less than 2%. “In” and “Off” represent cotton-growing season and off-season samples, respectively. “Live” and “Heat killed” represent live sclerotia and heat-killed sclerotia samples, respectively.


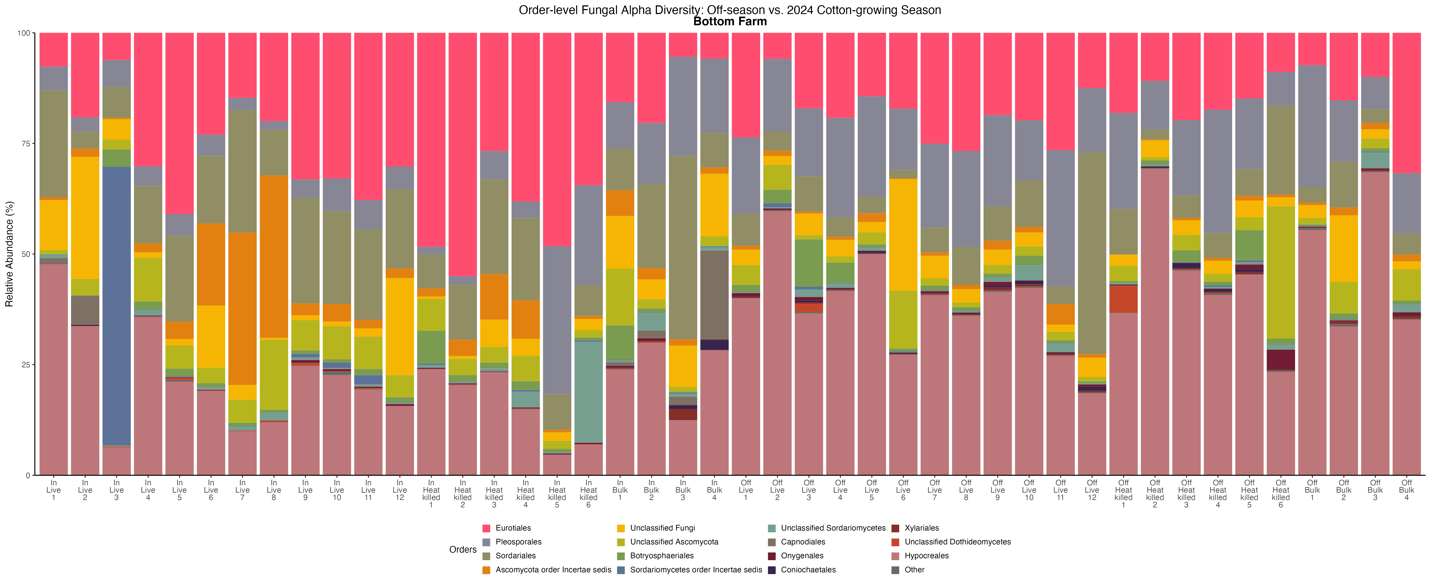


**A**

**B**


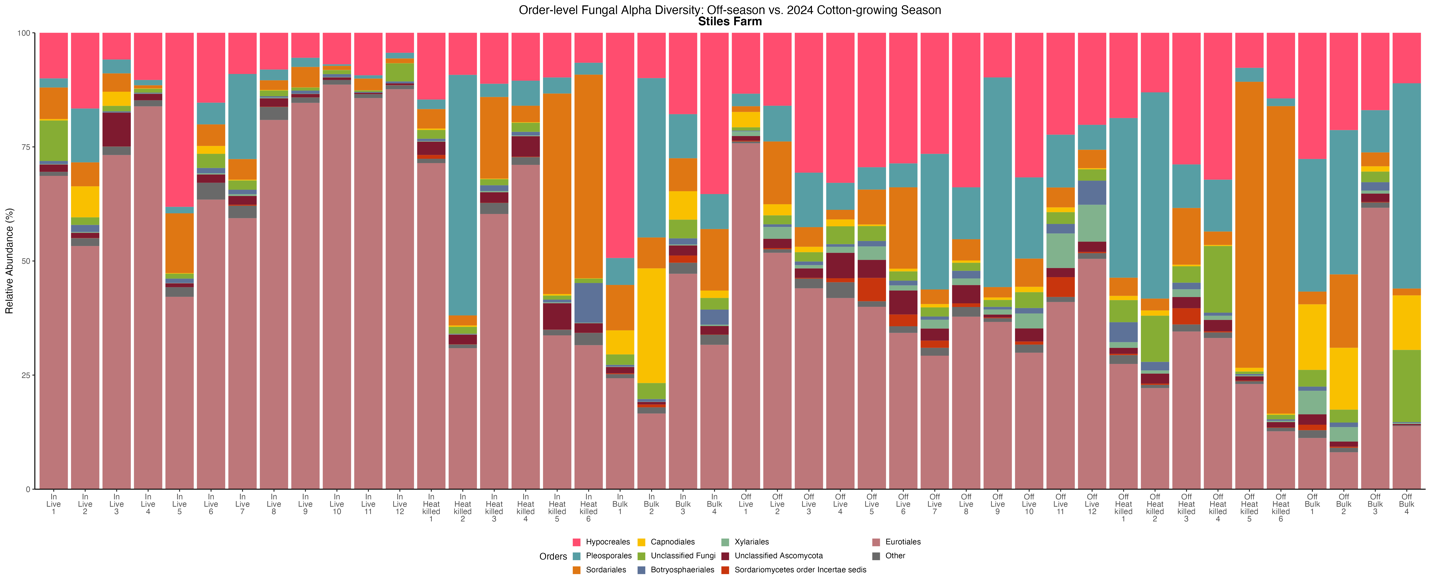


**Supplementary Figure 10.** Stacked bar charts showing fungal diversity of all samples, at both locations, during the off-season and 2024 cotton-growing season. **(A)** Bottom Farm. Orders classified as “Other” were found at a relative abundance less than 2%. **(B)** Stiles Farm. Orders classified as “Other” were found at a relative abundance less than 2.5%. “In” and “Off” represent cotton-growing season and off-season samples, respectively. “Live” and “Heat killed” represent live sclerotia and heat-killed sclerotia samples, respectively.

**2 Supplementary Tables**

**Supplementary Table 1.** Average relative abundance (%) for the most abundant bacterial orders in the 2023-2024 off-season and the 2024 cotton-growing season, as well as the comparison between seasons at the Bottom and Stiles farms.

*See Supplementary_abundance_bacterial_tables.xlsx*

**Supplementary Table 2.** Average relative abundance (%) for the most abundant fungal orders in the 2023-2024 off-season and the 2024 cotton-growing season, as well as the comparison between seasons at the Bottom and Stiles farms.

*See Supplementary_abundance_fungal_tables.xlsx*

**Supplementary Table 3.** Change over three seasons at the Stiles Farm in live sclerotia-associated bacterial communities. Within an order, average relative abundance values with the same letter are not significantly different.

*See sheet “Live”*

**Supplementary Table 3.** Change over three seasons at the Stiles Farm in heat-killed sclerotia-associated bacterial communities. Within an order, average relative abundance values with the same letter are not significantly different.

*See sheet “Heat-killed”*

**Supplementary Table 3.** Change over three seasons at the Stiles Farm in bulk soil-associated bacterial communities. Within an order, average relative abundance values with the same letter are not significantly different.

*See sheet “Bulk”*

**Supplementary Table 4.** Change over three seasons at the Stiles Farm in live sclerotia-associated fungal communities. Within an order, average relative abundance values with the same letter are not significantly different.

*See sheet “Live”*

**Supplementary Table 4.** Change over three seasons at the Stiles Farm in heat-killed sclerotia-associated fungal communities. Within an order, average relative abundance values with the same letter are not significantly different.

*See “Heat-killed”*

**Supplementary Table 4.** Change over three seasons at the Stiles Farm in bulk soil-associated fungal communities. Within an order, average relative abundance values with the same letter are not significantly different.

*See sheet “Bulk”*
